# Comparative Mitogenomic Analysis of Five Awl Skippers (Lepidoptera: Hesperiidae: Coeliadinae) and Their Phylogenetic Implications

**DOI:** 10.3390/insects12080757

**Published:** 2021-08-23

**Authors:** Qi Sun, Yumeng Yang, Xiangyu Hao, Jintian Xiao, Jiaqi Liu, Xiangqun Yuan

**Affiliations:** 1 College of Life Sciences, Northwest A&F University, Yangling 712100, China; qsun@nwafu.edu.cn (Q.S.); xyhao@nwafu.edu.cn (X.H.); 2 College of Natural Resources and Environment, Northwest A&F University, Yangling 712100, China; Yangyumeng@nwafu.edu.cn; 3 Key Laboratory of Plant Protection Resources and Pest Management, Ministry of Education, College of Plant Protection, Northwest A&F University, Yangling 712100, China; xjt0629@nwafu.edu.cn (J.X.); jiaq_work@163.com (J.L.)

**Keywords:** mitochondrial genome, mitogenome, phylogeny

## Abstract

**Simple Summary:**

The subfamily Coeliadinae (Lepidoptera: Hesperiidae) is a unique group of over 70 species in the butterfly family, and its mitochondrial genome data still needs to be supplemented. This study sequenced and analyzed five additional complete mitochondrial genomes of the Coeliadinae species (*Hasora schoenherr*, *Burara miracula*, *B*. *oedipodea*, *B*. *harisa*, and *Badamia exclamationis*) and compared them in detail with those of the other known skipper mitogenomes. All five of these mitogenomes have the typical lepidopteran mitogenome characteristics of 13 protein-coding genes, 22 transfer RNA genes, 2 ribosomal RNA genes, and a non-coding region. Our results indicate that their structure, nucleotide composition, codon usage, secondary structure of tRNAs, and so on, are highly conserved. Expanded sampling and gene data from the GenBank, phylogenetic analyses using maximum likelihood, and Bayesian inference methods indicate that Coeliadinae is monophyletic. These results contribute toward refining the phylogeny.

**Abstract:**

To determine the significance of mitochondrial genome characteristics in revealing phylogenetic relationships and to shed light on the molecular evolution of the Coeliadinae species, the complete mitochondrial genomes (mitogenomes) of five Coeliadinae species were newly sequenced and analyzed, including *Hasora schoenherr*, *Burara miracula*, *B*. *oedipodea*, *B*. *harisa*, and *Badamia exclamationis*. The results show that all five mitogenomes are double-strand circular DNA molecules, with lengths of 15,340 bp, 15,295 bp, 15,304 bp, 15,295 bp, and 15,289 bp, respectively, and contain the typical 37 genes and a control region. Most protein-coding genes (PCGs) begin with ATN, with 3 types of stop codons including TAA, TAG, and an incomplete codon T-; most of the genes terminate with TAA. All of the transfer RNA genes (tRNAs) present the typical cloverleaf secondary structure except for the *trnS1*. Several conserved structural elements are found in the AT-rich region. Phylogenetic analyses based on three datasets (PCGs, PRT, and 12PRT) and using maximum likelihood (ML) and Bayesian inference (BI) methods show strong support for the monophyly of Coeliadinae, and the relationships of the five species are (*B*. *exclamationis* + ((*B*. *harisa* + (*B*. *oedipodea* + *B*. *miracula*)) + *H*. *schoenherr*)).

## 1. Introduction

The insect mitochondrial genome (mitogenome) is a double-strand circular DNA molecule, regularly containing 13 protein-coding genes (PCGs), 2 ribosomal RNAs (rRNAs), 22 transfer RNAs (tRNAs), and an AT-rich region. The molecular data in mitochondria have been widely used in molecular taxonomy and phylogenetic analysis of different levels in Insecta [1] because of their rapid evolution, relatively conserved genes [1,2], and the negligible impact of gene flow (Zhang et al. noted that the ancient gene flow consistently caused erroneous reconstruction of the phylogeny [3]). So far, only five available mitogenome sequences and three batches of unassembled Sequence Read Archive (SRA) raw data from Coeliadinae can be accessed in the GenBank. Therefore, in this study, we sequenced five species of the subfamily Coeliadinae to support mitochondrial genomics research and we analyzed their phylogenetic relationships.

Hesperiidae, commonly known as skippers, are a major group of butterflies, many of which are agricultural pests, often appearing in farmlands and shrubs. The latest systematic research shows that the family Hesperiidae is divided into 13 subfamilies: Coeliadinae, Euschemoninae, Eudaminae, Tagiadinae, Pyrrhopyginae, Pyrginae, Katreinae, Chamundinae, Heteropterinae, Malazinae, Barcinae, Trapezitinae, and Hesperiinae [4,5]. Hao et al. [6] suggested two options: (1) To combine Pyrginae, Eudaminae, and Tagiadinae as Pyrginae *sensu lato*, and to combine Barcinae, Trapezitinae, and Hesperiinae as Hesperiinae *sensu lato*. (2) To divide them into separate subfamilies as suggested by Zhang et al. [4], with the subfamily Eudaminae retained. To simplify our analysis and highlight our key group Coeliadinae, we temporarily choose the former scheme.

The subfamily Coeliadinae, commonly named awls or policemen skippers due to the shape of their labial palpi and their characteristic fast-flying chase [7], are a small taxon of over 70 species located at the basal lineage among the Hesperiidae [8,9,10,11]. The Coeliadinae are beautifully colored skippers which are medium to large in size [12]. They are only distributed in the Old World from Western equatorial Africa to Yemen and Socotra, from western India to Japan, and from China to Australia and across most Pacific archipelagos [7,13]. A number of species of Coeliadinae were successively described after Drury (1773) [14] reported the first species of this subfamily.

## 2. Materials and Methods

### 2.1. Sample Collection and DNA Extraction

The adult specimens of *Hasora schoenherr* (Latreille, 1824) were collected in Mengla County, Xishuangbanna, Yunnan Province, China, in August 2019. The *Burara miracula* (Evans, 1949) were sampled in the Nanling National Forest Park, Shaoguan, Guangdong Province, China, in July 2020. *Burara oedipodea* (Swainson, 1820), *B*. *harisa* (Moore, 1866), and *Badamia exclamationis* (Fabricius, 1775) were collected in the Cuc Phuong National Park, Ninh Binh, Vietnam, in July 2019. All fresh specimens were immediately preserved in absolute ethanol and stored at −80 °C in the Entomological Museum of Northwest A & F University, Yangling, Shaanxi, China. The total DNA was extracted from thoracic tissue using the EasyPure^®^ Genomic DNA Kit (TransGen Biotech, Beijing, China). We used only one adult sample of breast muscle tissue of each species to extract DNA.

### 2.2. Sequencing, Assembly, Annotation and Bioinformatic Analyses

The entire mitochondrial genomes of the five adult individuals were sequenced separately by next-generation sequencing (NGS) on an Illumina HiSeq 4000 platform (Biomarker Technologies, Beijing, China). Approximately 1.2 GB of clean data from each species were used to assemble de novo into circular genomes using CLC Genomics Workbench v10.0.1 (CLC Bio, Aarhus, Denmark). Gene features were annotated on the Geneious 8.1.3 software (Biomatters, Auckland, New Zealand) following work with *B*. *striata* (Hewitson, 1867) [15]. The ORFs of the 13 protein-coding genes were predicted based on the invertebrate mitochondrial genetic code Table 5. The position and structure of 22 tRNAs and 2 rRNAs were predicted using the MITOS Web Server [16], and were then drawn in Adobe Illustrator. Tandem repeats of the AT rich region were identified with the Tandem Repeats Finder server (http://tandem.bu.edu/trf/trf.html) (28 June 2020) [17]. Finally, the order and orientation of all genes were checked on Geneious and compared to available sequences from the GenBank. In addition, the SRA raw data (*Tekliades ramanatek*, *Bibasis mahintha*, and *Hasora chromus* with DNA Voucher NVG-7871, NVG-7865, and NVG-17119G11, respectively) were download from the GenBank and assembled using the same methods above.

The circular maps of mitogenomes were produced by the CGView Server [18]. The nucleotide composition and relative synonymous codon usage (RSCU) of these five mitogenomes and their related species were calculated with PhyloSuite [19]. The AT skew and CG skew were calculated with the following formulas: AT − skew = [A − T]/[A + T] and GC − skew = [G − C]/[G + C] [20]. All five mitogenomes (*H. schoenherr*, *B. miracula*, *B. oedipodea*, *B. harisa*, and *B. exclamationis*) have already been uploaded to the GenBank with the Accession Numbers MZ502493, MZ502491, MZ502492, MZ502490, and MZ502489, respectively.

### 2.3. Phylogenetic Analysis

We selected five newly determined species of Coeliadinae and 36 available mitogenomes of related taxa (including all available coeliad sequences and the representative species of each genus in other subfamilies) from the GenBank to explore the status of Coeliadinae in the family Hesperiidae and its relationship with other subfamilies (Table 1). Four papilionid butterflies, *Parnassius apollo* (Linnaeus, 1758), *Graphium timur* (Ney, 1911), *Papilio machaon* (Linnaeus, 1758), and *P*. *helenus* (Linnaeus, 1758), were selected as the outgroups (Table 1). The phylogenetic relationships were reconstructed based on three datasets: (1) 13 protein-coding genes (PCGs); (2) 13 protein-coding genes + 2 rRNAs + 22 tRNAs (PRT); and (3) First and second codon positions of 13 protein-coding genes + 2 rRNAs + 22 tRNAs (12PRT).

The PCGs and RNAs were first extracted with PhyloSuite. Multiple sequences of PCGs and RNAs were then aligned using the G-INS-i and Q-INS-i strategies in MAFFT [21], respectively. The ambiguously aligned sites were removed using the Gblocks software [22]. The multiple sequences of each species were then concatenated using concatenate sequences and converted into Nexus and Phylip formats in PhyloSuite. The PartitionFinder V2.1.1 [23] was used to select the optimal partitioning scheme and nucleotide substitution model for the evolutionary tree (Tables S3 and S4). The maximum likelihood (ML) method and Bayesian inference (BI) method were used for a phylogenetic analysis based on three datasets.

The ML tree was reconstructed by IQ-TREE [24], and the support value for each node was evaluated by the standard bootstrap (BS) algorithm, which was tested 50,000 times. The BI tree was reconstructed by MrBayes v3.2.6 [25]. Two independent runs were run for 10 million generations, and samples were taken every 1000 generations. Four independent Markov Chains (including three heated chains and a cold chain) were run. A consensus tree was obtained from all the trees after the initial 25% of trees from each MCMC run were discarded as burn-in, with the chain convergence assumed after the average standard deviation of split frequencies fell below 0.01.

## 3. Results and Discussion

### 3.1. Structure and Nucleotide Composition of Mitogenome

**Figure 1:**
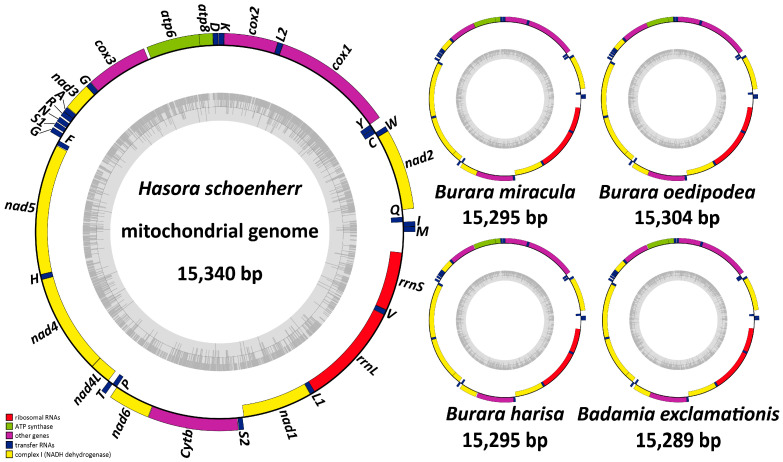
Circular maps of mitogenomes of five species in Coeliadinae.

The total lengths of the mitogenomes of *H. schoenherr*, *B. miracula*, *B. oedipodea*, *B. harisa*, and *B. exclamationis* are 15,340 bp, 15,295 bp, 15,304 bp, 15,295 bp, and 15,289 bp, respectively (Figure 1, Table S1). The five complete mitogenomes show similar nucleotide compositions with 39.3% A, 40.7% T, 12.2% C, and 7.8% G in *H. schoenherr*; 39.8% A, 41% T, 11.7% C, and 7.4% G in *B. miracula*; 39.5% A, 40.7% T, 12% C, and 7.7% G in *B. oedipodea*; 39.4% A, 40.9% T, 12.1% C, and 7.7% G in *B. harisa*; and 39.5% A, 40.9% T, 11.8% C, and 7.8% G in *B. exclamationis*. All the genomes exhibit a strong base composition bias toward A + T, ranging from 80% to 80.8% in five species (mean value = 80.34%). The third position of the codon in PCGs has a higher A + T content than the position of the first and second positions. The A + T content in RNAs is also higher than that of PCGs. The AT nucleotide content of the 13 PCGs is the lowest, with a mean content of 78.74% among the above five species, which is consistent with known lepidopteran insects [6,15,26]. Moreover, all mitogenomes have a slightly negative AT-skew (ranging from −0.019 to −0.015, mean = −0.0172) and a negative GC-skew (ranging from −0.223 to −0.203, mean = −0.2172) (Table S1).

### 3.2. Protein-Coding Genes and Codon Usage

**Figure 2:**
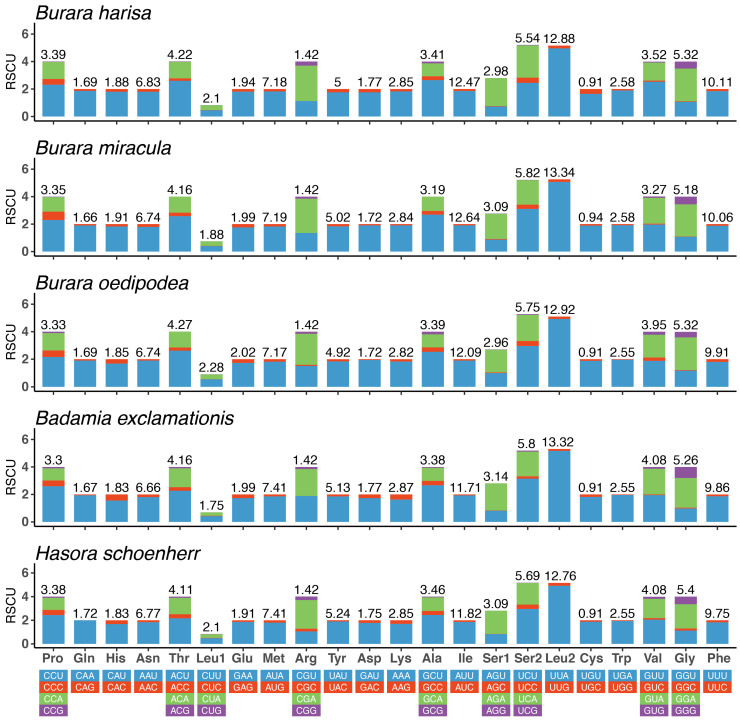
Relative synonymous codon usage (RSCU) in the mitogenomes of five Coeliadinae species.

The total lengths of 13 the PCGs of *H. schoenherr*, *B. miracula*, *B. oedipodea*, *B. harisa*, and *B. exclamationis* are 11,196 bp, 11,205 bp, 11,193 bp, 11,187 bp, and 11,199 bp, respectively. Of the 13 PCGs, the smallest gene is the *atp8*, and the largest gene is the *nad5*, ranging from 162 bp to 1743 bp in *B. miracula* and *B. exclamationis*, respectively (Table S2). The five species mentioned above have negative AT-skews (mean = −0.1582) and positive GC-skews (mean = 0.0156) in PCGs (Table S1). Across the 13 PCGs, only four PCGs (*nad1*, *nad4*, *nad4L*, and *nad5*) are encoded on the N-strand, whereas the other nine (*cox1*, *cox2*, *cox3*, *atp6*, *atp8*, *nad2*, *nad3*, *nad6*, and *Cytb*) are located on the J-strand. Almost all PCGs in the five species start with the standard codon ATN (ATC, ATG, ATT, and ATA), except for *cox1* in *H. schoenherr*, *B. oedipodea*, *B. harisa*, and *B. exclamationis*, using the CGA as the start codon [31], while *cox1* in *B. miracula* starts with ATT. Stop codons in the PCGs include three types: TAA, TAG, or T. Most stop codons are TAA, except *nad3* of *B. miracula* and *B. oedipodea*, and *nad2* of *B. harisa*, which stop with the termination codon TAG, while an incomplete stop codon T- was found in the *cox1*, *cox2*, *nad4*, and *nad5* in *H. schoenherr*, *B. miracula*, *B. oedipodea*, and *B. harisa,* and *cox1*, *cox2*, and *nad4* in *B. exclamationis.* Therefore, the occurrence of the termination codon TAA is more common than TAG, and at least three incomplete stop codons and T-codons are present in all five mitogenomes (Table S2). Statistics on the relative synonymous codon usage (RSCU) of the five skippers show that the codon UUA (Leu2) is used most frequently (Figure 2).

### 3.3. Transfer and Ribosomal RNA Genes

**Figure 3:**
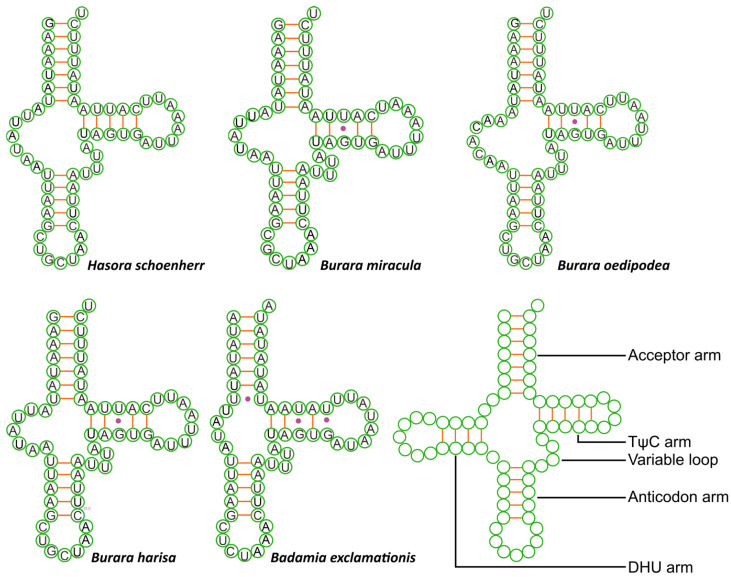
Predicted secondary cloverleaf structure of *trnS1* (AGN) of five species. Typical structure of tRNA is shown below right.

The total lengths of the 22 tRNAs of the five species range between 1458 bp (*H. schoenherr*) and 1470 bp (*B. harisa*), with an average value of 1464 bp, which is consistent with typical Lepidoptera species [6,15,26]. The 22 tRNA sizes range from 61 bp (*trnS1*) to 71 bp (*trnK*) in *H. schoenherr*, from 61 bp (*trnS1*) to 72 bp (*trnD*) in *B. miracula*, from 61 bp (*trnS*) to 71 bp (*trnK*) in *B. oedipodea* and *B. harisa*, and from 58 bp (*trnS1*) to 71 bp (*trnK*) in *B. exclamationis* (Table S2). All tRNAs can be folded into a typical cloverleaf structure except for *trnS1* (Figure 3). Due to the limited length of this manuscript, the remaining tRNA secondary structures are presented in the supplementary materials (Figures S6–S10). Most of the 22 tRNAs in these mitogenomes demonstrate a positive AT-skew and GC-skew, except for the identical content of the A and T found in *B. exclamationis* (Table S1).

The two rRNA genes (*rrnS* and *rrnL*) are encoded on the N-strand in these species. The large rRNA (*rrnL*) located between *trnL1* and *trnV* ranges in length from 1367 bp (*B. oedipodea* and *B. harisa*) to 1403 bp (*H. schoenherr*), while the small rRNA (*rrnS*) located between *trnV* and the AT-rich region ranges in length from 772 bp (*B. exclamationis*) to 804 bp (*B. oedipodea*) (Table S2). The two rRNAs with a typical lepidopteran AT nucleotide bias [6,15,26] range from 84.5% (*B. harisa*) to 85.0% (*B. exclamationis*) (mean = 84.74%) (Table S1).

### 3.4. Overlapping Sequences, Intergenic Spacers and AT-Rich Region

There are five gene overlaps in *H. schoenherr*, ranging in size from 2 bp to 21 bp, and seven gene overlaps in *B. exclamationis*, ranging from 2 bp to 19 bp, while 10 gene overlaps appear in the *B. miracula* mitogenome and six gene overlaps occur in the *B. oedipodea* and *B. harisa* mitogenomes with a size from 1 to 21 bp. The longest overlap region of the five mitogenomes is located between *trnL1* and *rrnL*. All five species have two identical overlap regions, including *trnW*-*trnC* (8 bp) and *atp8*-*atp6* (7 bp) (Table S2).

In *H. schoenherr*, *B. miracula*, *B. oedipodea*, *B. harisa*, and *B. exclamationis*, 19, 15, 21, 18, and 16 intergenic spacers are observed, respectively, with a size from 1 to 91 bp, 1 to 104 bp, 1 to 90 bp, 1 to 86 bp, and 1 to 108 bp, respectively, with the longest intergenic spacer being located between *trnQ* and *nad2*. Three intergenic spacers are common to all five mitogenomes, including *trnP*-*nad6* (2 bp), *trnS2*-*nad1* (17 bp), and *nad1*-*trnL1* (1 bp). The lengths of the intergenic spacers are more variable than the number of overlaps (Table S2).

The AT-rich region, also named the control region, is the largest non-coding region in the mitogenome and is responsible for the origin of transcription and replication [45,46]. This region, the sizes of which in the five species ranges from 262 bp (*B. exclamationis*) to 285 bp (*B. miracula*), is located between *rrnS* and *trnM*. Like typical lepidopteran insects [6,15,26], the A + T contents of the control region are the highest in mitogenomes with the mean value 93.84% (Table S1).

**Figure 4:**
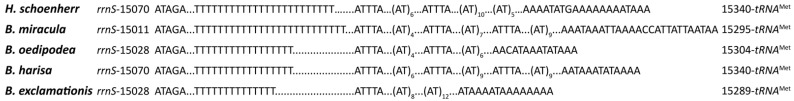
Structural elements found in the AT-rich region of five skippers (The presented nucleotides indicate the conserved sequences, dots between sequences indicate omitted sequences).

All of the five AT-rich regions exist with dinucleotide repeats with the (AT) type. Except for *B. exclamationis* and *B.oedipodea*, which have two short dinucleotide repeats ((AT)_8_ and (AT)_12_) and ((AT)_4_ and (AT)_6_), respectively, the other three species all have three dinucleotide repeats of small size, ranging from (AT)_4_ to (AT)_10_. Our results suggest that the motif ‘ATAGA,’ followed by a long poly-T stretch, ranging from 14 bp (*B. exclamationis*) to 26 bp (*B. miracula*), is the origin of the light-strand replication which is close to the 5′-end of *rrnS*. Several microsatellite-like sequences following the motif ATTTA are found in the five skipper mitogenomes (Figure 4).

### 3.5. Phylogenetic Relationships

**Figure 5:**
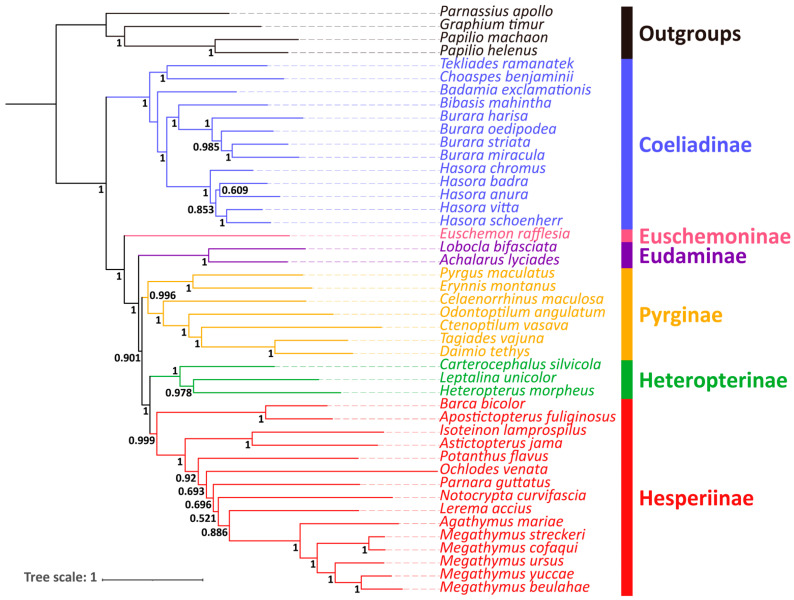
Phylogenetic relationships inferred by the BI method based on the PRT dataset. Numbers on nodes are the posterior probabilities (PP).

In this study, five newly determined species and 41 available sequences (including four papilionids as outgroups) on the GenBank were used to reconstruct the phylogenetic relationships of the subfamily Coeliadinae and its related taxa. The relationships among Coeliadinae were slightly different based on two analyses (ML and BI) of three different datasets (PCG, PRT, and 12PRT). The relationships in the genus *Burara* are *B. oedipodea* + (*B. miracula* + (*B. striata* + *B. harisa*)) in the two trees based on the 12PRT dataset, while in the other trees, the relationship is *B. harisa* + (*B. oedipodea* + (*B. striata* + *B. miracula*)). We selected the tree with the highest support values (PRT_BI) as the phylogenetic hypothesis in this article (Figure 5); the others are in the Supplementary Materials (Figures S1–S5). The bootstrap support values, BS, and the posterior probability, PP, are shown in the branch nodes of the ML and BI trees, respectively.

Our results indicate that the phylogenetic positions of the subfamilies in Hesperiidae are (Coeliadinae + (Euschemoninae + (Eudaminae + (Pyrginae + (Heteropterinae + Hesperiinae))))), with most branches receiving a high support value and confirming the monophyly of the six main subfamilies. In order to highlight the focused taxon (Coeliadinae) and simplify the analysis, the Pyrginae and Hesperiinae included here are all Pyrginae *sensu lato* and Hesperiinae *sensu lato* as suggested by Hao et al. [6]. The five species we mainly focused on are all members of Coeliadinae, which is the basal lineage among Hesperiidae, and is strongly supported (PP = 1) as a sister to the remaining subfamilies.

The relationships of inter-genus within Coeliadinae are ((*Tekliades* + *Choaspes*) + (*Badamia* + ((*Bibasis* + *Burara*) + *Hasora*))). Our analyses demonstrate consistent support for the monophyly of Coeliadinae similar to previous studies [4,11,13], while its internal topologies are different in severally aspects. In the phylogenetic tree, *Tekliades ramanatek* (Boisduval, 1833) and *Choaspes benjaminii* (Guérin-Meneville, 1843) form a clade with high support value (PP = 1) and a sister to all remaining Coeliadinae, including *B. exclamationis*, whereas Toussaint’s study [13] showed that *B. exclamationis* is a sister to the grouping of *T. ramanatek* + *C. benjaminii*, and the three species mentioned above are clustered into one clade in relation to the rest of Coeliadinae. The phylogenetic relationships among *Hasora* are also not in line with previous studies. Our results show the relationship is (*H. chromus* + ((*H. badra* + *H. anura*) + (*H. vitta* + *H. schoenherr*))), which is discordant with Toussaint et al.’s result [13], ((*H. badra* + *H. anura*) + (*H. chromus* + (*H. schoenherr* + *H. vitta*))). The topological incongruence might be the result of incomplete lineage sorting [47,48,49] or the mito-nuclear conflict [48].

## 4. Conclusions

In this study, we newly sequenced five complete mitogenomes to describe the characteristics of genomes and align phylogenetic relationships with their related taxa. After our comparisons to other complete mitogenomes of Coeliadinae, the species *H*. *schoenherr*, *B*. *miracula*, *B*. *oedipodea*, *B*. *harisa*, and *B*. *exclamationis*, were found to be consistent in structure, nucleotide composition, protein-coding genes, and codon usage, etc., and were highly conserved, in agreement with previous studies. Bayesian inference and maximum likelihood phylogenetic analyses based on the three datasets (PCGs, PRT, and 12PRT) produce well-resolved topologies, with most branches having strong support. We recovered Coeliadinae as monophyletic with high support, and the relationship we determined was (*Badamia exclamationis* + ((*Burara harisa* + (*Burara oedipodea* + *Burara miracula*)) + *Hasora schoenherr*)), we also revealed the new relationships of *Hasora*, which were (*Hasora schoenherr* + (*Hasora chromus* + (*Hasora badra* + (*Hasora vitta* + *Hasora anura*)))), which may contribute to resolving the phylogeny and provide valuable additional data.

## Figures and Tables

**Table 1:** Classification and origins of the mitochondrial genome used in this study.

Taxon	Species	Accession Number/ DNA Voucher	References
Hesperiidae			
Coeliadinae	*Burara miracula*	MZ502491	This study
	*Burara oedipodea*	MZ502492	This study
	*Burara harisa*	MZ502490	This study
	*Burara striata*	NC_034676	[15]
	*Badamia exclamationis*	MZ502489	This study
	*Tekliades ramanatek*	NVG-7871	[4]
	*Bibasis mahintha*	NVG-7865	[4]
	*Choaspes benjaminii*	NC_024647	[26]
	*Hasora chromus*	NVG-17119G11	[4]
	*Hasora anura*	KF881049	[27]
	*Hasora vitta*	NC_027170	[28]
	*Hasora badra*	NC_045249	Unpublished
	*Hasora schoenherr*	MZ502493	This study
Euschemoninae	*Euschemon rafflesia*	NC_034231	[29]
Pyrginae	*Celaenorrhinus maculosus*	NC_022853	[30]
	*Ctenoptilum vasava*	JF713818	[31]
	*Tagiades (*=*Daimio) Tethys*	KJ813807	[32]
	*Erynnis montanus*	NC_021427	[33]
	*Pyrgus maculatus*	NC_030192	Unpublished
	*Tagiades vajuna*	KX865091	[34]
	*Odontoptilum angulatum*	MW381783	[35]
Eudaminae	*Achalarus lyciades*	NC_030602	[36]
	*Lobocla bifasciata*	KJ629166	[26]
Heteropterinae	*Carterocephalus silvicola*	NC_024646	[26]
	*Heteropterus morpheus*	NC_028506	Unpublished
	*Leptalina unicolour*	MK265705	[37]
Hesperiinae	*Apostictopterus fuliginosus*	NC_039946	[38]
	*Barca bicolor*	NC_039947	[38]
	*Lerema accius*	NC_029826	[39]
	*Ochlodes venata*	HM243593	Unpublished
	*Parnara guttatus*	NC_029136	[40]
	*Potanthus flavus*	KJ629167	[26]
	*Astictopterus jama*	MH763663	[41]
	*Isoteinon lamprospilus*	MH763664	[41]
	*Notocrypta curvifascia*	MH763665	[41]
	*Agathymus mariae*	KY630504	[15]
	*Megathymus beulahae*	KY630505	[15]
	*Megathymus cofaqui*	KY630503	[15]
	*Megathymus streckeri*	KY630501	[15]
	*Megathymus ursus*	KY630502	[15]
	*Megathymus yuccae*	KY630500	[15]
Outgroup			
Papilionidae	*Papilio machaon*	NC_018047	Unpublished
	*Papilio helenus*	NC_025757	[42]
	*Graphium timur*	NC_024098	[43]
	*Parnassius apollo*	NC_024727	[44]

## Data Availability

The following information was supplied regarding the availability of DNA sequences: The complete mitogenomes of *H. schoenherr*, *B. miracula*, *B. oedipodea*, *B. harisa*, and *B. exclamationis* have already been uploaded to the GenBank with the Accession Numbers MZ502493, MZ502491, MZ502492, MZ502490, and MZ502489, respectively.

## Supplementary Materials

The following are available online at https://www.mdpi.com/article/10.3390/insects12080757/s1, Figure S1: Phylogenetic tree inferred by ML method based on PRT dataset. Numbers on nodes are the bootstrap support values (BS), Figure S2: Phylogenetic tree inferred by ML method based on PCG dataset. Numbers on nodes are the bootstrap support values (BS), Figure S3: Phylogenetic tree inferred by ML method based on 12PRT dataset. Numbers on nodes are the bootstrap support values (BS), Figure S4: Phylogenetic tree inferred by BI method based on PCG dataset. Numbers on nodes are the posterior probabilities (PP), Figure S5: Phylogenetic tree inferred by BI method based on 12PRT dataset. Numbers on nodes are the posterior probabilities (PP), Figure S6: Predicted secondary cloverleaf structure of tRNA of *H. schoenherr*, Figure S7: Predicted secondary cloverleaf structure of tRNA of *B. miracula*, Figure S8: Predicted secondary cloverleaf structure of tRNA of *B. oedipodea*, Figure S9: Predicted secondary cloverleaf structure of tRNA of *B. harisa*, Figure S10: Predicted secondary cloverleaf structure of tRNA of *B. exclamationis*, Table S1: Nucleotide composition and skewness of different elements of mitogenomes of *H. schoenherr*, *B. miracula*, *B. oedipodea*, *B. harisa* and *B. exclamationis*, Table S2: Mitogenomic organization of *H. schoenherr*, *B. miracula*, *B. oedipodea*, *B. harisa* and *B. exclamationis*, Table S3: Best partitioning schemes and models based on different datasets for BI analysis, Table S4: Best partitioning schemes and models based on different datasets for ML analysis.
