# Supplementary material for: Comparative Mitogenomic Analysis of Five Awl Skippers (Lepidoptera: Hesperiidae: Coeliadinae) and Their Phylogenetic Implications
Source: Insects. 2021 Aug 23;12(8):757. doi: 10.3390/insects12080757 (PMC8397065; doi:10.3390/insects12080757)
Supplement: Supplementary file 1 [file insects-12-00757-s001.zip › Supplementary Materials.pdf]

Supplementary Materials

Comparative Mitogenomic Analysis of Five Awl Skippers (Lepidoptera: Hesperiidae: Coeliadinae) and Their Phylogenetic Implications

Qi Sun [1,4], Yumeng Yang [2,4], Xiangyu Hao [1,4], Jintian Xiao [3,4], Jiaqi Liu [3,4] and Xiangqun Yuan [3,*]

[1] College of Life Sciences, Northwest A&F University, Yangling 712100, China; qsun@nwafu.edu.cn (Q. S.); xyhao@nwafu.edn.cn (X. H.)
[2] College of Natural Resources and Environment, Northwest A&F University, Yangling 712100, China; Yangyumeng@mwafu.edu.cn (Y. Y.)
[3] Key Laboratory of Plant Protection Resources and Pest Management, Ministry of Education; College of Plant Protection, Northwest A&F University, Yangling 712100, China; xjt0629@nwafu.edu.cn (J. X.); jiaq_work@163.com (J. L.)
[4] These authors contributed equally to this article.
** Correspondence: yuanxq@nwsuaf.edu.cn; Tel.: +86-1375-998-5152

Table S1. Nucleotide composition and skewness of different elements of mitogenomes of *H. schoenherr*, *B. miracula*, *B. oedipodea*, *B. harisa* and *B. exclamationis*.

Regions	Size (bp)	T(U)%	C%	A%	G%	A+T%	AT skew	GC skew
				H. schoenherr/B. miracula/B. oedipodea/B. harisa/B. exclamationis				
PCGs	11196/11205/11193/11187/11199	45.2/46.1/45.7/45.4/45.6	10.7/10.3/10.6/10.6/10.2	33.0/33.3/33.0/33.3/33.1	11.1/10.4/10.8/10.7/11.0	78.2/79.4/78.7/78.7/78.7	-0.156/-0.161/-0.161/-0.154/-0.159	0.021/0.005/0.010/0.003/0.039
1st codon position	3732/3735/3731/3729/3733	37.1/37.9/37.1/37.2/37.7	10.4/10.2/10.6/10.5/9.9	36.0/36.6/36.0/36.4/35.9	16.6/15.3/16.3/15.9/16.4	73.1/74.5/73.1/73.6/73.6	-0.015/-0.018/-0.016/-0.010/-0.025	0.227/0.200/0.215/0.207/0.247
2nd codon position	3732/3735/3731/3729/3733	47.8/48.3/48.2/48.1/48.0	16.6/16.5/16.7/16.5/16.6	22.2/22.1/22.0/22.2/22.1	13.3/13.2/13.1/13.2/13.3	70.0/70.4/70.2/70.3/70.1	-0.365/-0.373/-0.374/-0.370/-0.369	-0.109/-0.112/-0.119/-0.112/-0.112
3rd codon position	3732/3735/3731/3729/3733	50.8/52.0/51.6/51.0/51.2	5.0/4.1/4.5/4.8/4.0	40.8/41.3/41.0/41.3/41.4	3.5/2.6/2.9/2.9/3.3	91.6/93.3/92.6/92.3/92.6	-0.108/-0.115/-0.115/-0.105/-0.106	-0.178/-0.219/-0.215/-0.254/-0.094
NCR	271/285/277/278/262	48.3/49.8/48.4/48.2/47.7	4.8/2.8/5.1/4.0/4.6	44.6/46.0/44.0/46.0/46.2	2.2/1.4/2.5/1.8/1.5	92.9/95.8/92.4/94.2/93.9	-0.040/-0.040/-0.047/-0.023/-0.016	-0.368/-0.333/-0.333/-0.375/-0.500
*trnA*s	1458/1464/1468/1470/1460	39.9/40.0/39.4/40.3/40.7	7.8/7.9/8.0/8.1/7.7	41.2/41.5/41.8/40.5/40.7	11.0/10.7/10.8/11.0/10.9	81.1/81.5/81.2/80.8/81.4	0.016/0.018/0.029/0.003/0.000	0.171/0.154/0.148/0.153/0.169
rRNAs	2186/2157/2171/2144/2166	41.1/40.9/41.3/40.0/41.3	4.9/4.9/5.3/5.2/4.9	43.5/44.0/43.4/44.5/43.7	10.6/10.2/10.0/10.3/10.2	84.6/84.9/84.7/84.5/85.0	0.028/0.036/0.024/0.053/0.028	0.367/0.354/0.313/0.331/0.350
Full genome	15340/15295/15304/15295/15289	40.7/41.0/40.7/40.9/40.9	12.2/11.7/12.0/12.1/11.8	39.3/39.8/39.5/39.4/39.5	7.8/7.4/7.7/7.7/7.8	80.0/80.8/80.2/80.3/80.4	-0.018/-0.015/-0.016/-0.019/-0.018	-0.219/-0.221/-0.220/-0.223/-0.203

Table S2. Mitogenomic organization of *H. schoenherr, B. miracula, B. oedipodea, B. harisa* and *B. exclamationis.*

Gene	Position		Size	Intergenic nucleotides	Codon		Strand
	From	To			Start	Stop	
			H. schoenherr/B. miracula/B. oedipodea/B. harisa/B. exclamationis				
trnM	1/1/1/1/1	68/68/69/69/68	68/68/69/69/68				J
trnI	69/69/72/70/71	132/135/137/137/135	64/67/66/68/65	-/-/2/-/2			J
trnQ	130/137/138/146/133	198/205/206/214/201	69/69/69/69/69	-3/1/-/8/-3			N
nad2	290/310/297/301/310	1303/1323/1310/1314/1323	1014/1014/1014/1014/1014	91/104/90/86/108	ATT/ATT/ATT/ATT/ATT	TAA/TAA/TAA/TAG/TAA	J
trnW	1302/1322/1309/1328/1322	1368/1388/1375/1394/1389	67/67/67/67/68	-2/-2/-2/13/-2			J
trnC	1361/1381/1368/1387/1382	1426/1449/1433/1451/1446	66/69/66/65/65	-8/-8/-8/-8/-8			N
trnY	1428/1468/1449/1466/1449	1494/1532/1514/1533/1515	67/65/66/68/67	1/18/15/14/2			N
cox1	1497/1530/1521/1538/1530	3027/3066/3051/3068/3060	1531/1537/1531/1531/1531	2/-3/6/4/14	CGA/ATT/CGA/CGA/CGA	T/T/T/T/T	J
trnL2	3028/3067/3052/3069/3061	3094/3133/3118/3135/3127	67/67/67/67/67				J
cox2	3095/3136/3120/3136/3128	3776/3817/3801/3817/3809	682/682/682/682/682	-/2/1/-/-	ATG/ATG/ATG/ATG/ATG	T/T/T/T/T	J
trnK	3777/3818/3802/3818/3810	3847/3888/3872/3888/3880	71/71/71/71/71				J
trnD	3852/3894/3877/3894/3893	3917/3965/3943/3960/3959	66/72/67/67/67	4/5/4/5/12			J
atp8	3918/3966/3944/3961/3960	4094/4127/4108/4125/4127	177/162/165/165/168		ATC/ATT/ATT/ATT/ATT	TAA/TAA/TAA/TAA/TAA	J
atp6	4088/4121/4102/4119/4121	4765/4798/4779/4796/4798	678/678/678/678/678	-7/-7/-7/-7/-7	ATG/ATG/ATG/ATG/ATG	TAA/TAA/TAA/TAA/TAA	J
cox3	4803/4798/4786/4811/4815	5588/5601/5583/5599/5600	786/804/798/789/786	37/-1/6/14/16	ATG/ATA/ATT/ATG/ATG	TAA/TAA/TAA/TAA/TAA	J
trnG	5591/5605/5586/5602/5603	5658/5672/5653/5667/5668	68/68/68/66/66	2/3/2/2/2			J
nad3	5659/5673/5654/5668/5669	6012/6026/6007/6021/6022	354/354/354/354/354		ATT/ATT/ATT/ATT/ATC	TAA/TAG/TAG/TAA/TAA	J
trnA	6016/6025/6006/6024/6025	6079/6089/6072/6088/6089	64/65/67/65/65	3/-2/-2/2/2			J
trnR	6080/6091/6075/6088/6090	6142/6154/6138/6155/6153	63/64/64/68/64	-/1/2/-1/-			J
trnN	6162/6168/6146/6156/6154	6227/6233/6211/6221/6219	66/66/66/66/66	19/13/7/-/-			J
trnS1	6237/6242/6226/6238/6233	6297/6302/6286/6298/6290	61/61/61/61/58	9/8/14/16/13			J
trnE	6316/6350/6321/6335/6322	6381/6415/6386/6402/6388	66/66/66/68/67	18/47/34/36/31			J

Gene	Position		Size	Intergenic nucleotides	Codon		Strand
	From	To			Start	Stop	
trnF	6422/6414/6404/6401/6391	6487/6477/6470/6469/6457	66/64/67/69/67	40/-2/17/-2/2			N
nad5	6495/6478/6472/6471/6458	8232/8215/8209/8205/8200	1738/1738/1738/1735/1743	7/-/1/1/-	ATT/ATT/ATT/ATT/ATT	T/T/T/T/TAA	N
trnH	8233/8216/8210/8206/8198	8299/8281/8276/8271/8263	67/66/67/66/66	-/-/-/-/-3			N
nad4	8300/8282/8277/8272/8264	9635/9617/9612/9607/9599	1336/1336/1336/1336/1336		ATG/ATG/ATG/ATG/ATG	T/T/T/T/T	N
nad4L	9636/9618/9612/9608/9600	9920/9902/9896/9892/9884	285/285/285/285/285	-/-/-1/-/-	ATG/ATG/ATG/ATG/ATG	TAA/TAA/TAA/TAA/TAA	N
trnT	9926/9908/9900/9897/9901	9991/9973/9965/9961/9966	66/66/66/65/66	5/5/3/4/16			J
trnP	9992/9974/9966/9962/9967	10057/10039/10031/10027/10032	66/66/66/66/66				N
nad6	10060/10042/10034/10030/10035	10593/10575/10564/10560/10568	534/534/531/531/534	2/2/2/2/2	ATT/ATT/ATT/ATC/ATT	TAA/TAA/TAA/TAA/TAA	J
cytb	10597/10575/10568/10588/10572	11742/11720/11713/11736/11723	1146/1146/1146/1149/1152	3/-1/3/27/3	ATG/ATG/ATG/ATG/ATG	TAA/TAA/TAA/TAA/TAA	J
trnS2	11747/11720/11718/11735/11722	11811/11785/11783/11799/11788	65/66/66/65/67	4/-1/4/-2/-2			J
nad1	11829/11803/11801/11817/11806	12767/12741/12739/12758/12744	939/939/939/942/939	17/17/17/17/17	ATG/ATG/ATG/ATG/ATG	TAA/TAA/TAA/TAA/TAA	N
trnL1	12769/12743/12741/12760/12746	12836/12809/12808/12827/12814	68/67/68/68/69	1/1/1/1/1			N
rrnL	12816/12789/12788/12807/12796	14218/14156/14154/14173/14189	1403/1368/1367/1367/1394	-21/-21/-21/-21/-19			N
trnV	14220/14158/14156/14175/14190	14286/14221/14223/14240/14255	67/64/68/66/66	1/1/1/1/-			N
rrnS	14287/14222/14224/14241/14256	15069/15010/15027/15017/15027	783/789/804/777/772				N
NCR	15070/15011/15028/15018/15028	15340/15295/15304/15295/15289	271/285/277/278/262				J

Table S3. Best partitioning schemes and models based on different datasets for BI analysis.

Dataset	Partitioning scheme	Models
PCGs	P1: (*cytb*_ pos1, *cox3*_ pos1, *atp6*_pos1)	GTR+I+G
	P2: (*cox1*_pos2, *cox3*_pos2, *cytb*_pos2, *cox2*_pos2, *atp6*_pos2)	GTR+I+G
	P3: (*cox3*_pos3, *nad3*_pos3, *nad6*_pos3, *cytb*_pos3, *cox2*_pos3, *atp6*_pos3, *cox1*_pos3, *atp8*_pos3)	GTR+G
	P4: (*atp8*_pos1, *nad3*_pos1, *nad6*_pos1, *atp8*_pos2)	GTR+I+G
	P5: (*cox2*_pos1, *cox1*_pos1)	GTR+I+G
	P6: (*nad1*_pos1, *nad5*_pos1, *nad4L*_pos1, *nad4*_pos1)	GTR+I+G
	P7: (*nad4L*_pos2, *nad4*_pos2, *nad5*_pos2, *nad1*_pos2)	GTR+I+G
	P8: (*nad4*_pos3, *nad1*_pos3)	GTR+G
	P9: (*nad6*_pos2, *nad3*_pos2, *nad2*_pos2)	GTR+I+G
	P10: (*nad2*_pos3)	GTR+G
	P11: (*nad4L*_pos3, *nad5*_pos3)	HKY+G
PRT	P1: (*cytb*_pos1, *cox3*_pos1, *atp6*_pos1)	GTR+I+G
	P2: (*cox1*_pos2, *cytb*_pos2, *cox3*_pos2, *cox2*_pos2, *atp6*_pos2)	GTR+I+G
	P3: (*nad6*_pos3, *atp6*_pos3, *cox1*_pos3, *atp8*_pos3, *cox2*_pos3, *nad3*_pos3, *cytb*_pos3, *cox3*_pos3)	GTR+G
	P4: (*nad6*_pos1, *atp8*_pos1)	GTR+I+G
	P5: (*trnE*, *atp8*_pos2, *trnR*)	F81+I+G
	P6: (*cox2*_pos1, *cox1*_pos1)	GTR+I+G
	P7: (*nad1*_pos1, *nad5*_pos1, *nad4*_pos1, *nad4L*_pos1)	GTR+I+G
	P8: (*nad4L*_pos2, *nad4*_pos2, *nad5*_pos2, *nad1*_pos2)	GTR+I+G
	P9: (*nad1*_pos3, *nad4*_pos3)	GTR+G
	P10: (*trnS2*, *trnT*, *trnI*, *trnS1*, *nad2*_pos1, *nad3*_pos1, *trnA*)	GTR+I+G
	P11: (*nad6*_pos2, *trnK*, *nad2*_pos2, *nad3*_pos2)	GTR+I+G
	P12: (*nad2*_pos3)	GTR+G
	P13: (*nad4L*_pos3, *nad5*_pos3)	HKY+G
	P14: (*rrnL*, *trnV*, *rrnS*, *trnY*)	GTR+I+G
	P15: (*trnQ*, *trnC*, *trnL1*)	HKY+G
	P16: (*trnD*, *trnH*, *trnF*, *trnP*)	HKY+I+G
	P17: (*trnG*, *trnW*, *trnM*, *trnL2*, *trnN*)	HKY+I+G
12PRT	P1: (*atp6*, *cox3*, *cytb*, *cox2*, *trnL2*)	GTR+I+G
	P2: (*nad6*, *atp8*)	GTR+I+G
	P3: (*cox1*)	GTR+I+G
	P4: (*nad4*, *nad1*, *nad5*)	GTR+I+G
	P5: (*trnS1*, *nad2*, *nad3*)	GTR+I+G
	P6: (*rrnL*, *trnV*, *trnY*, *nad4L*, *rrnS*)	GTR+I+G
	P7: (*trnR*, *trnN*, *trnA*, *trnS2*, *trnI*, *trnT*, *trnE*, *trnG*)	GTR+I+G
	P8: (*trnW*, *trnM*, *trnK*, *trnC*, *trnQ*, *trnL1*)	HKY+G
	P9: (*trnD*, *trnH*, *trnF*, *trnP*)	HKY+I+G

Table S4. Best partitioning schemes and models based on different datasets for ML analysis.

Datasets	Partitioning scheme	Models
PCGs	P1: (*cytb*_pos1, *atp6*_pos1, *cox3*_pos1)	GTR+I+G
	P2: (*cox3*_pos2, *cox2*_pos2, *atp6*_pos2, *cytb*_pos2, *cox1*_pos2)	TVM+I+G
	P3: (*nad6*_pos3, *atp6*_pos3, *cox2*_pos3, *cytb*_pos3)	TRN+G
	P4: (*atp8*_pos2, *atp8*_pos1)	K81UF+I+G
	P5: (*nad2*_pos3, *cox1*_pos3, *atp8*_pos3)	TIM+G
	P6: (*cox1*_pos1)	GTR+G
	P7: (*cox2*_pos1)	TRN+I+G
	P8: (*cox3*_pos3, *nad3*_pos3)	TRN+G
	P9: (*nad5*_pos1, *nad1*_pos1, *nad4*_pos1, *nad4L*_pos1)	GTR+I+G
	P10: (*nad4L*_pos2, *nad4*_pos2, *nad5*_pos2, *nad1*_pos2)	GTR+I+G
	P11: (*nad1*_pos3, *nad4*_pos3)	GTR+G
	P12: (*nad6*_pos1, *nad3*_pos1, *nad2*_pos1)	GTR+I+G
	P13: (*nad6*_pos2, *nad3*_pos2, *nad2*_po2)	TVM+I+G
	P14: (*nad4L*_pos3, *nad5*_pos3)	K81UF+I+G
PRT	P1: (*cytb*_pos1, *cox3*_pos1, *atp6*_pos1)	GTR+I+G
	P2: (*cox2*_pos2, *atp6*_pos2, *cox3*_pos2, *trnL2*, *cytb*_pos2, *cox1*_pos2)	TVM+I+G
	P3: (*nad6*_pos3, *atp8*_pos3, *cox2*_pos3, *atp6*_pos3)	TRN+G
	P4: (*atp8*_pos1, *atp8*_pos2, *trnE*)	K81UF+I+G
	P5: (*cox1*_pos1)	GTR+G
	P6: (*nad2*_pos3, *cox1*_pos3)	TIM+G
	P7: (*cox2*_pos1)	TRN+I+G
	P8: (*nad3*_pos3, *cox3*_pos3, *cytb*_pos3)	TRN+I+G
	P9: (*nad5*_pos1, *nad1*_pos1, *nad4*_pos1, *nad4L*_pos1)	GTR+I+G
	P10: (*nad4L*_pos2, *nad4*_pos2, *nad5*_pos2, *nad1*_pos2)	GTR+I+G
	P11: (*nad4L*_pos3, *nad5*_pos3, *nad1*_pos3, *nad4*_pos3)	TVM+I+G
	P12: (*nad6*_pos1, *nad2*_pos1, *trnS1*, *nad3*_pos1, *trnA*, *trnT*)	GTR+I+G
	P13: (*nad6*_pos2, *nad3*_pos2, *nad2*_pos2)	TVM+I+G
	P14: (*trnH*, *trnF*, *trnP*, *trnS2*, *rrnL*, *trnI*, *trnY*, *trnV*, *rrnS*)	GTR+I+G
	P15: (*trnC*, *trnK*, *trnQ*, *trnL1*)	HKY+G
	P16: (*trnW*, *trnN*, *trnM*, *trnG*, *trnD*, *trnR*)	K81UF+I+G
12PRT	P1: (*atp6*, *cox3*, *cytb*, *cox2*, *trnL2*)	GTR+I+G
	P2: (*nad6*, *atp8*)	GTR+I+G
	P3: (*cox1*)	GTR+I+G
	P4: (*nad4*, *nad1*, *nad5*)	TIM+I+G
	P5: (*trnS1*, *trnA*, *nad3*, *nad2*, *trnT*)	TIM+I+G
	P6: (*trnH*, *trnP*, rrnF, *rrnL*, *trnI*, *trnY*, *trnV*, *rrnS*, *nad4L*)	GTR+I+G
	P7: (*trnQ*, *trnL1*, *trnW*, *trnM*, *trnK*, *trnC*)	HKY+G

P8: (*trnG*, *trnE*, *trnN*, *trnR*, *trnD*, *trnS2*)	TIM+I+G

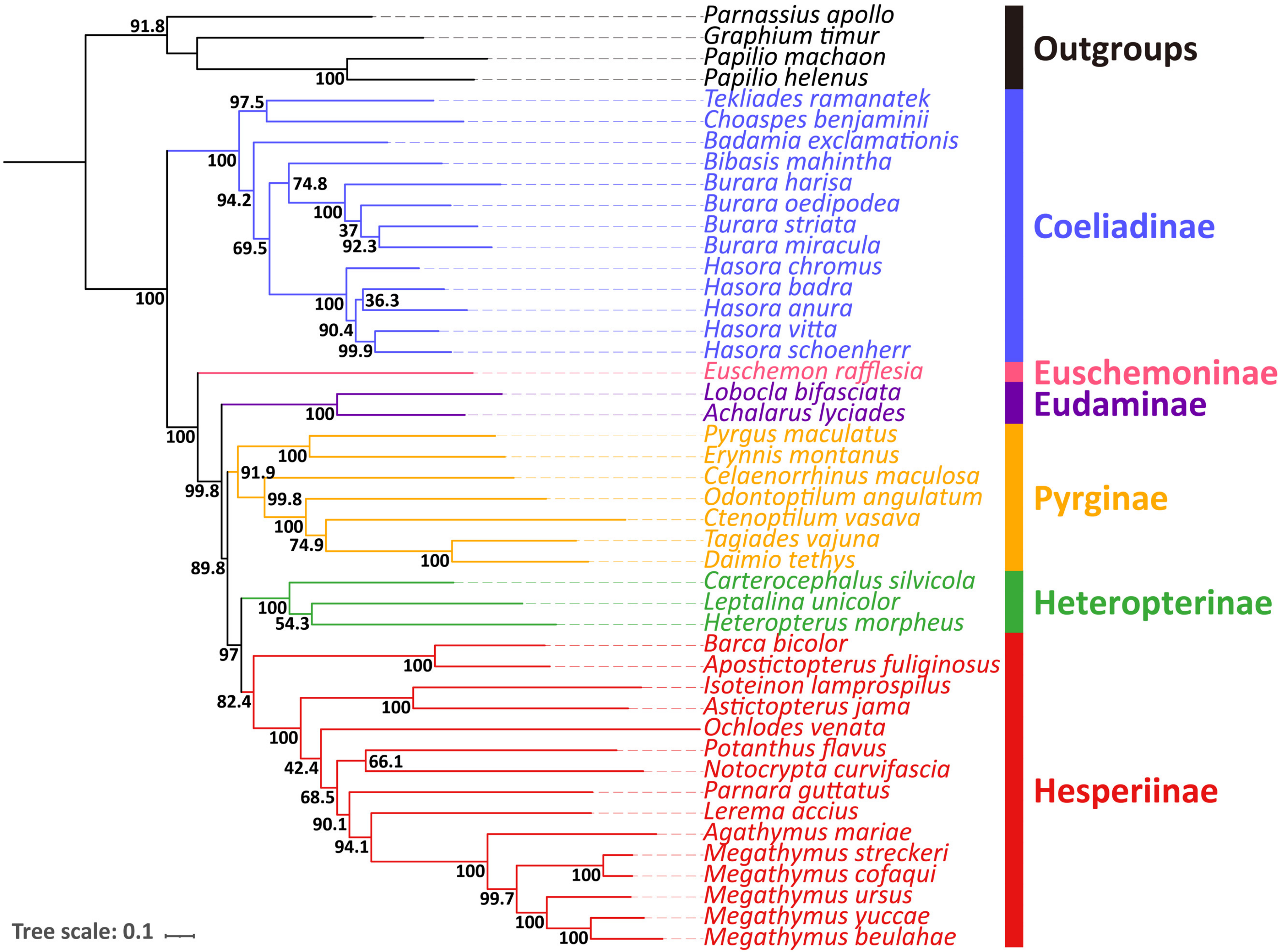

Figure S1. Phylogenetic tree inferred by ML method based on PRT dataset. Numbers on nodes are the bootstrap support values (BS).

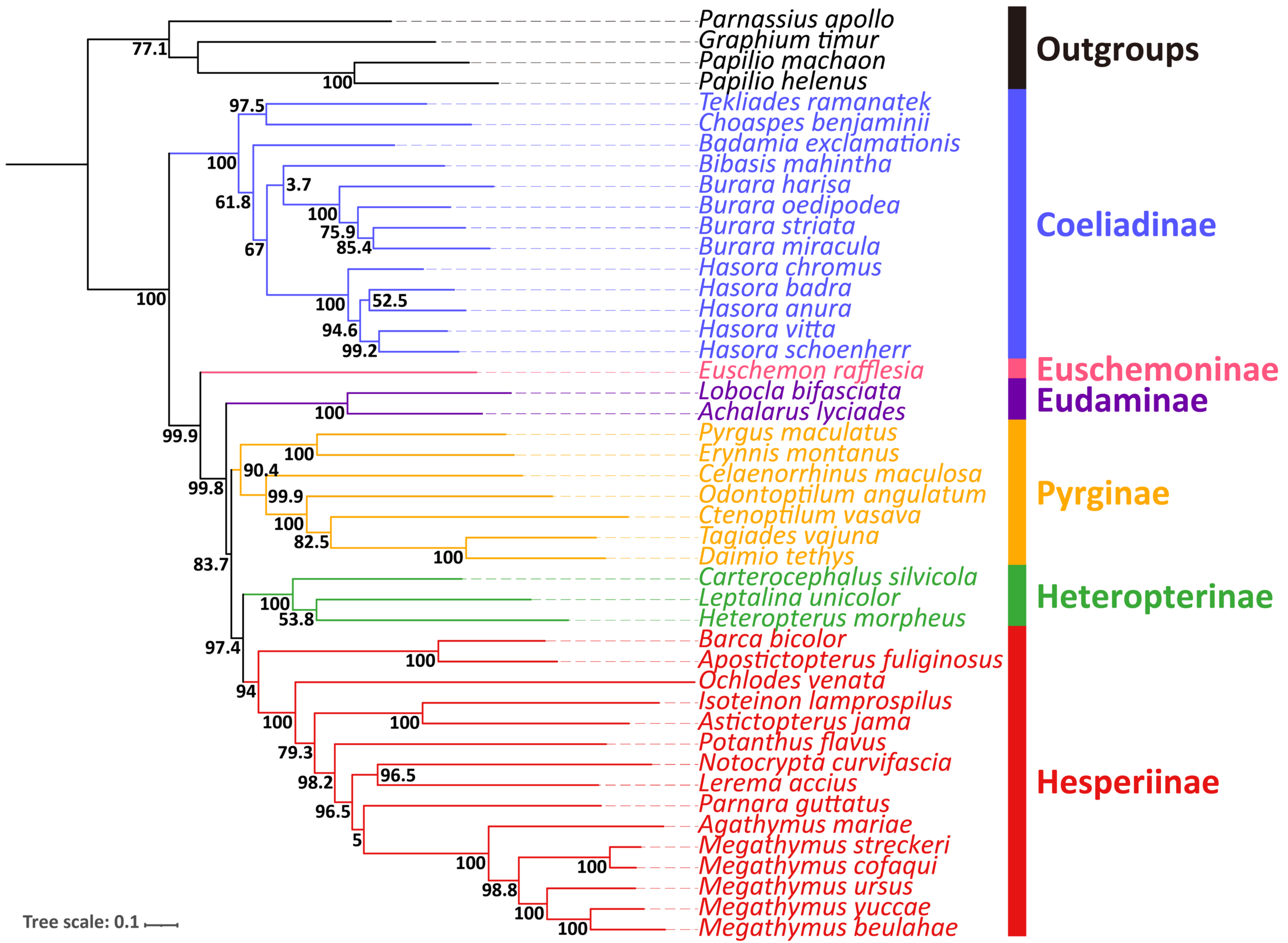

Figure S2. Phylogenetic tree inferred by ML method based on PCG dataset. Numbers on nodes are the bootstrap support values (BS).

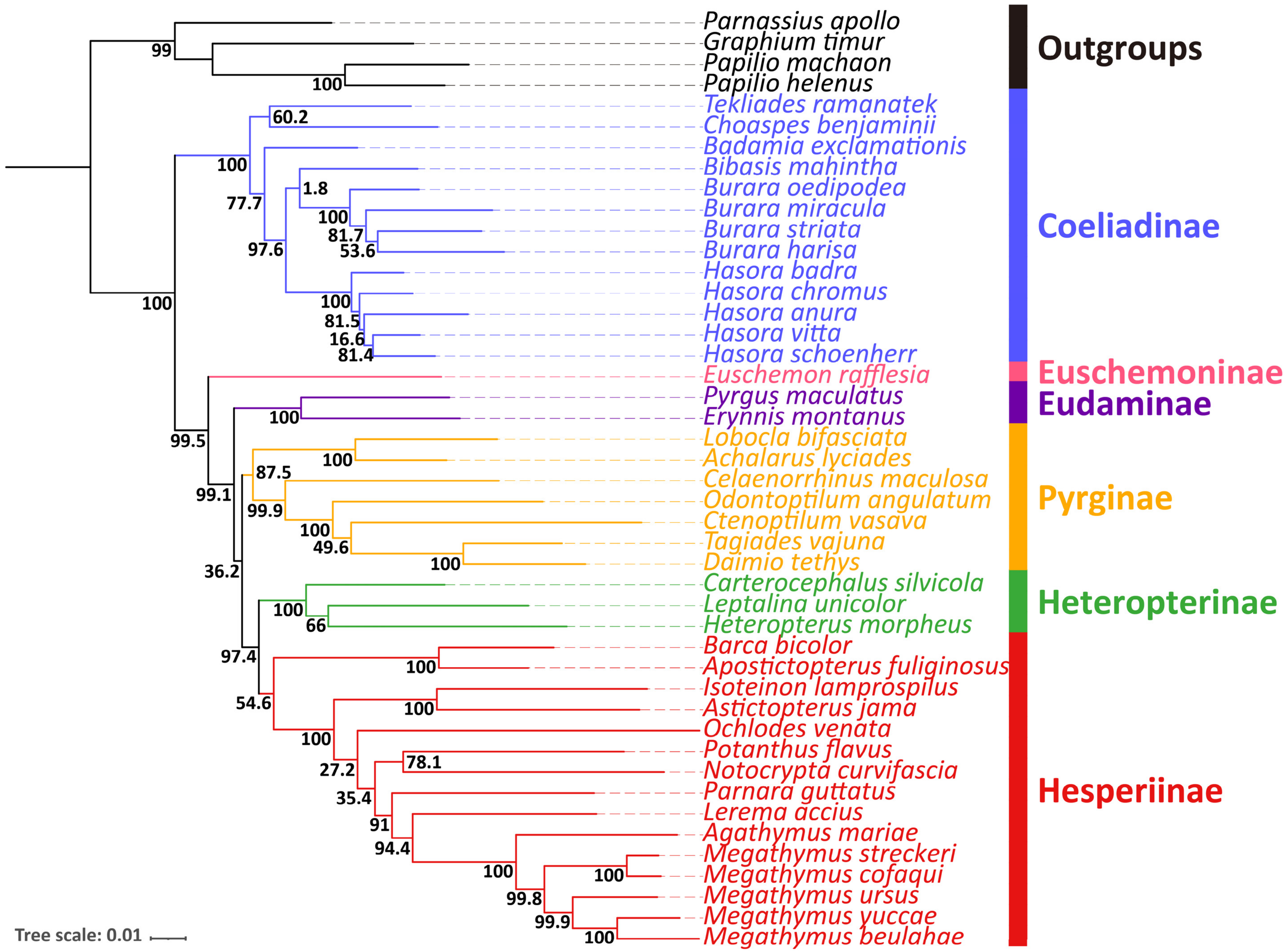

Figure S3. Phylogenetic tree inferred by ML method based on 12PRT dataset. Numbers on nodes are the bootstrap support values (BS).

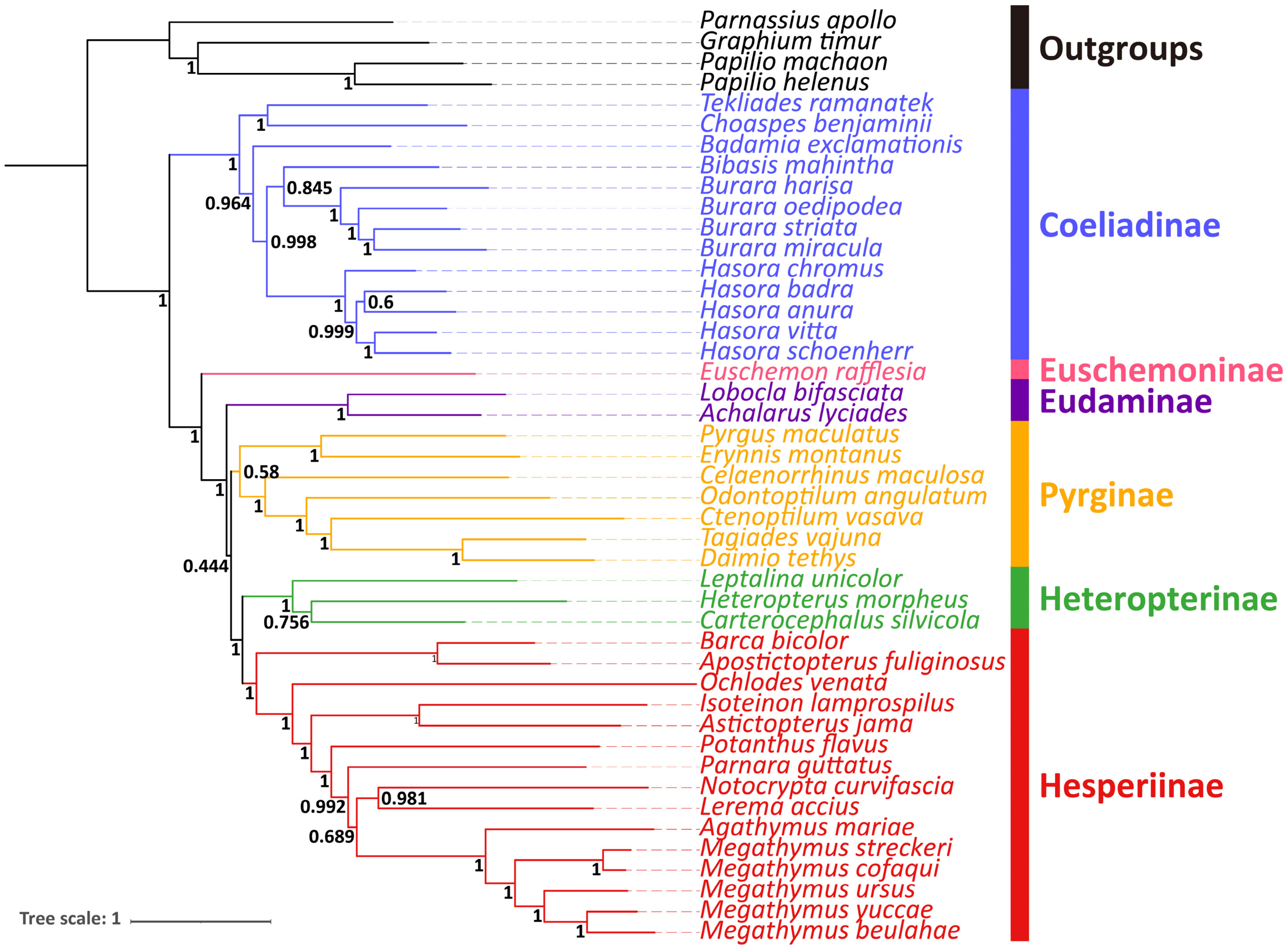

Figure S4. Phylogenetic tree inferred by BI method based on PCG dataset. Numbers on nodes are the posterior probabilities (PP).

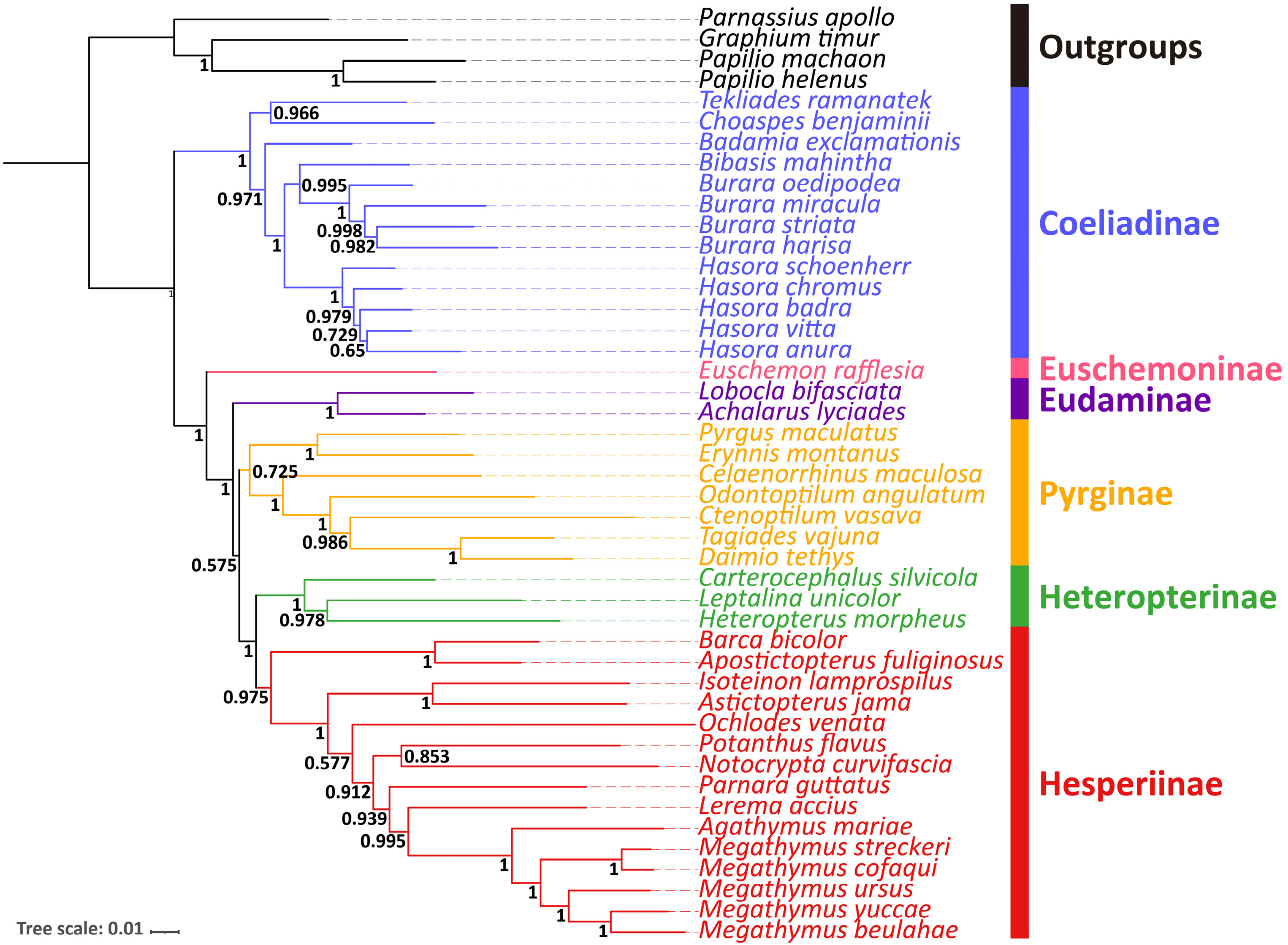

Figure S5. Phylogenetic tree inferred by BI method based on 12PRT dataset. Numbers on nodes are the posterior probabilities (PP).

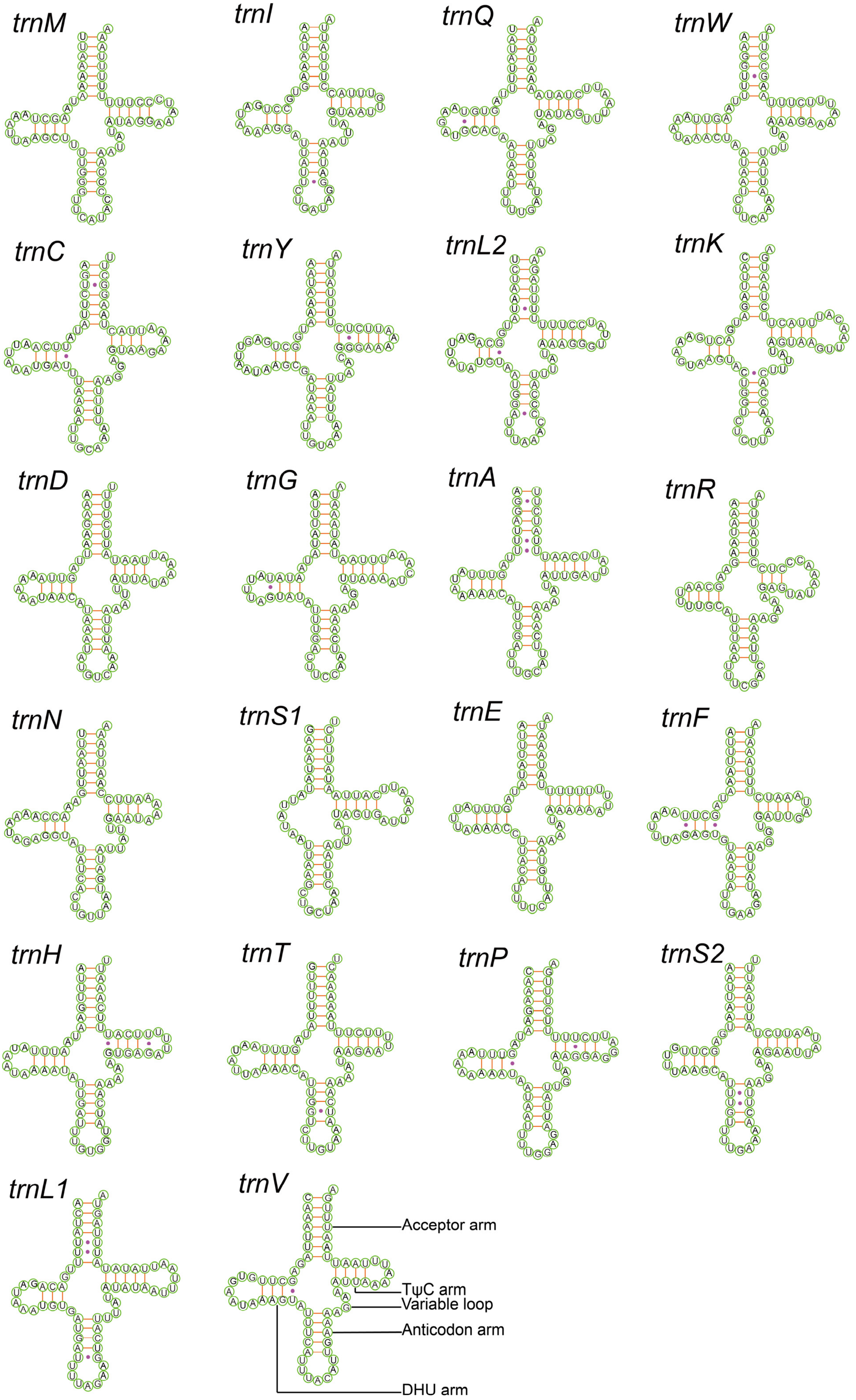

Figure S6. Predicted secondary cloverleaf structure of tRNA of *H. schoenherr*.

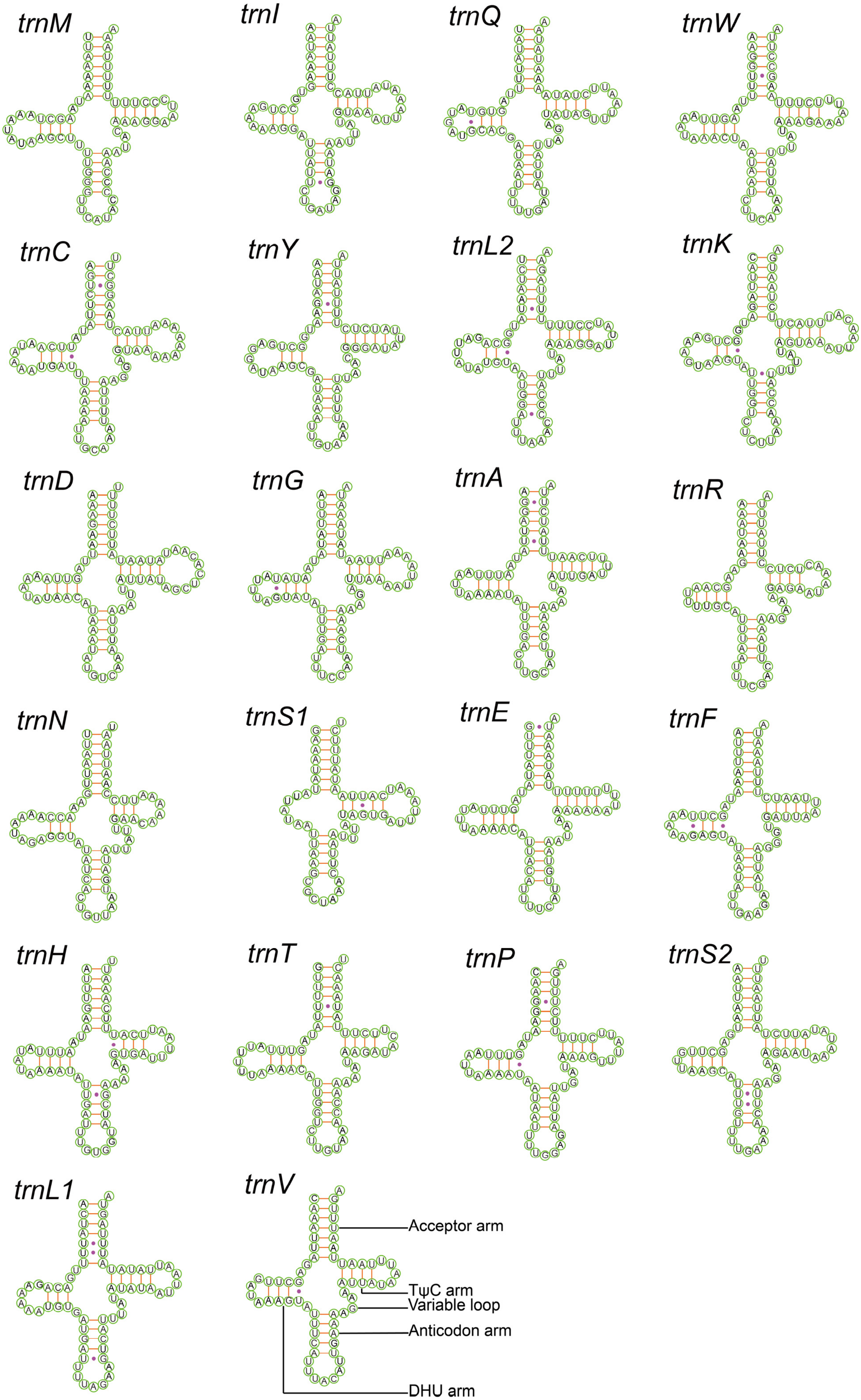

Figure S7. Predicted secondary cloverleaf structure of tRNA of *B. miracula*.

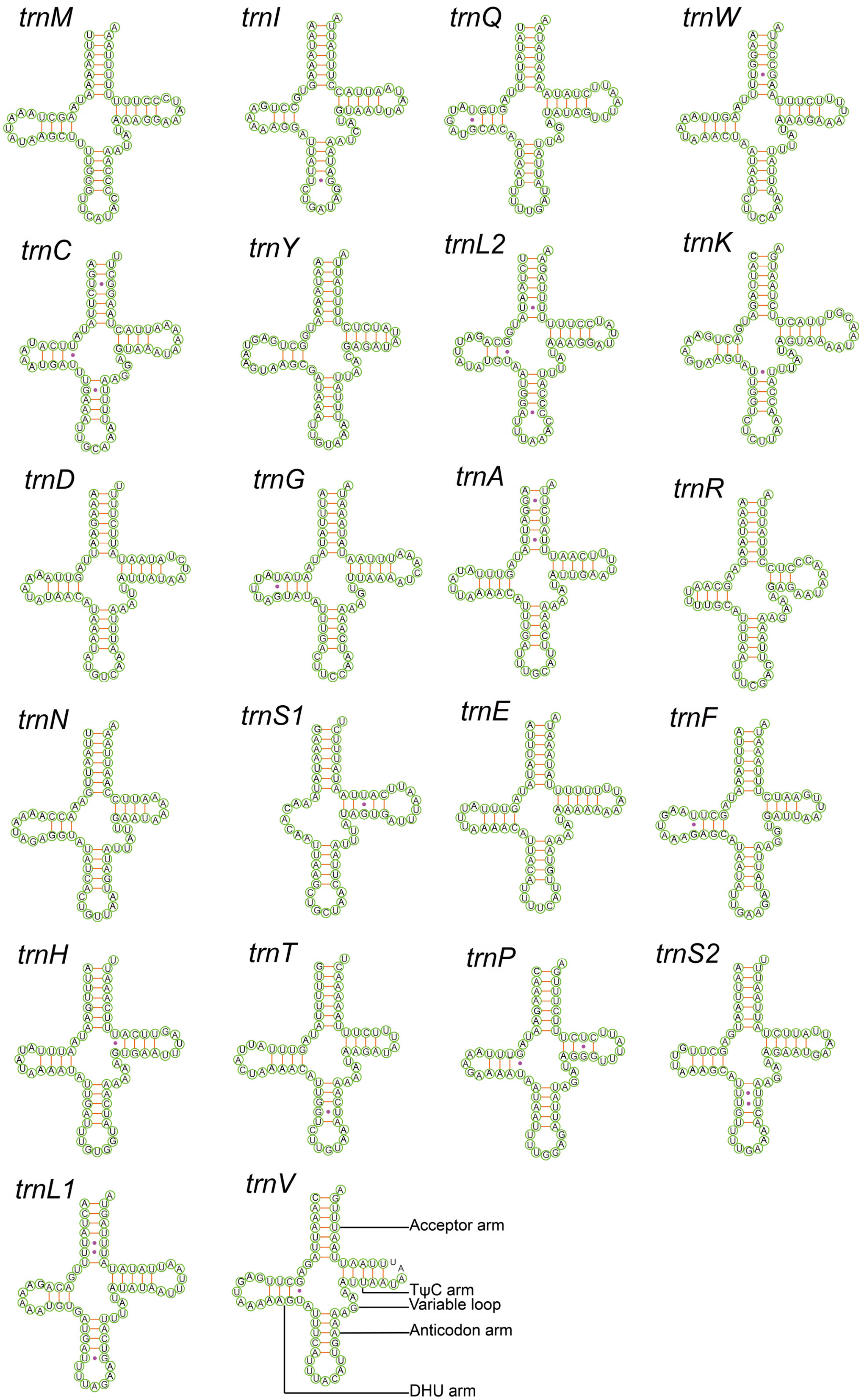

Figure S8. Predicted secondary cloverleaf structure of tRNA of *B. oedipodea*.

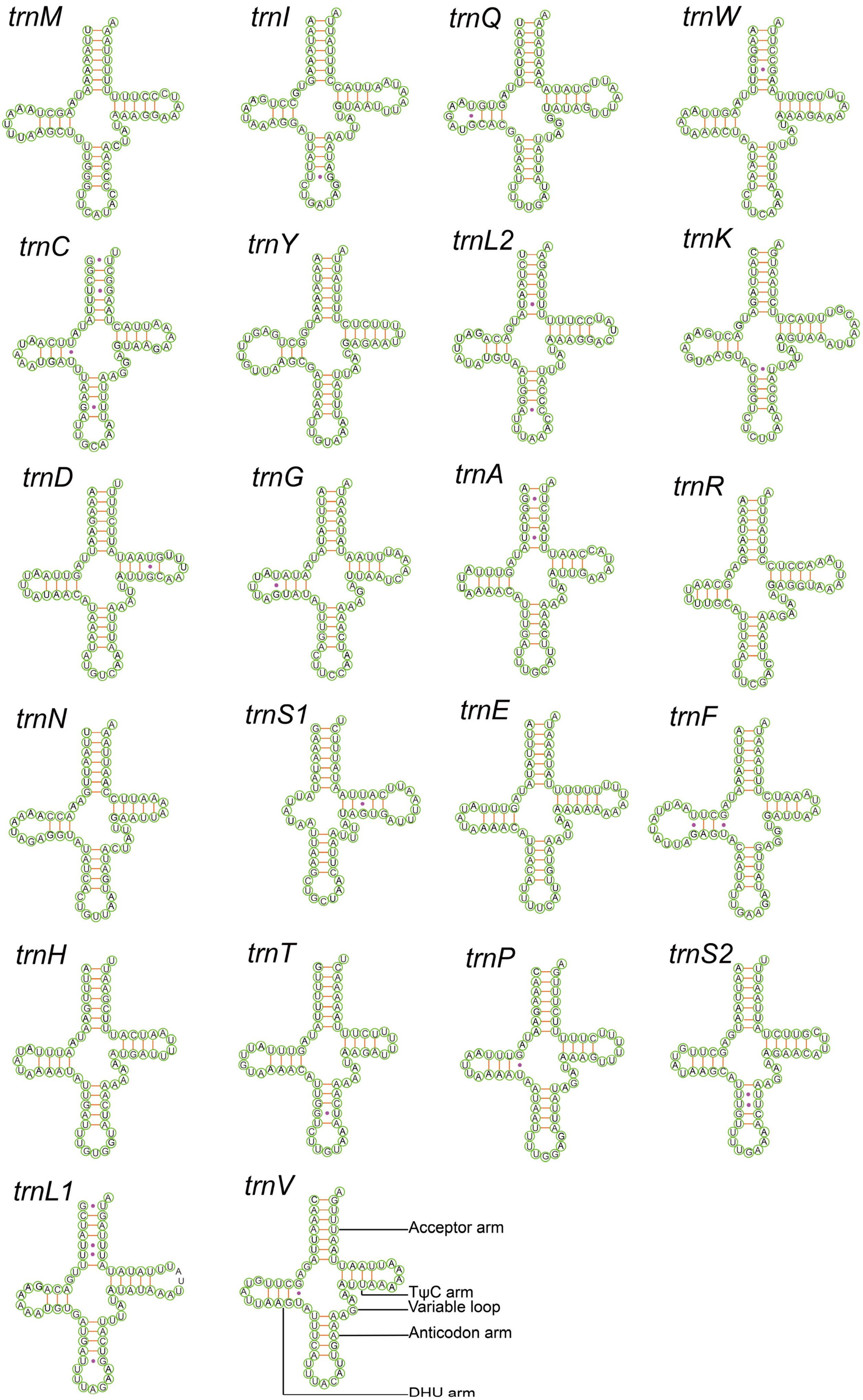

Figure S9. Predicted secondary cloverleaf structure of tRNA of *B. harisa*.

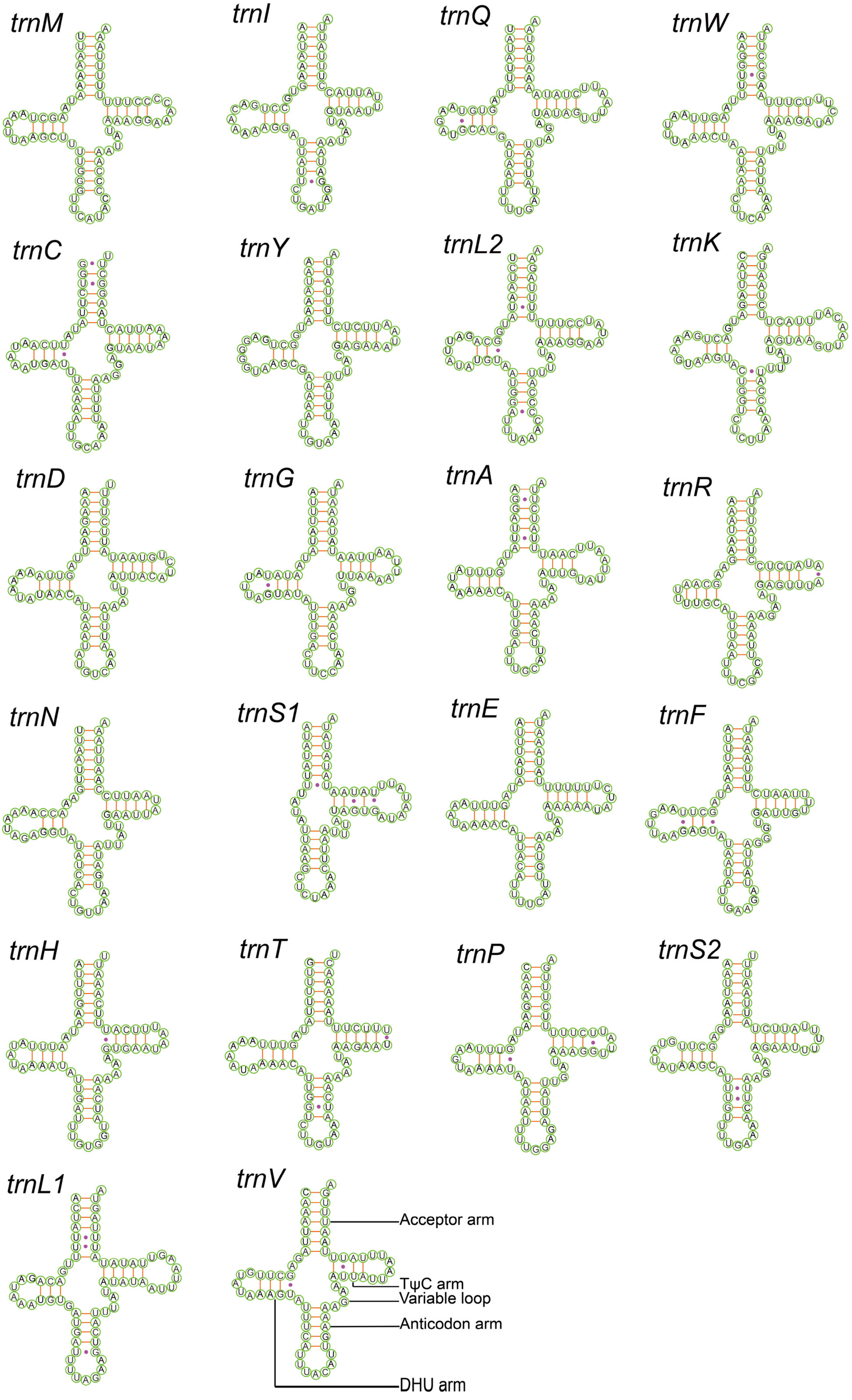

Figure S10. Predicted secondary cloverleaf structure of tRNA of *B. exclamationis*.
