# Supplementary figures and images for: Comparative Mitogenomic Analysis of Five Awl Skippers (Lepidoptera: Hesperiidae: Coeliadinae) and Their Phylogenetic Implications
Source: Insects. 2021 Aug 23;12(8):757. doi: 10.3390/insects12080757 (PMC8397065; doi:10.3390/insects12080757)

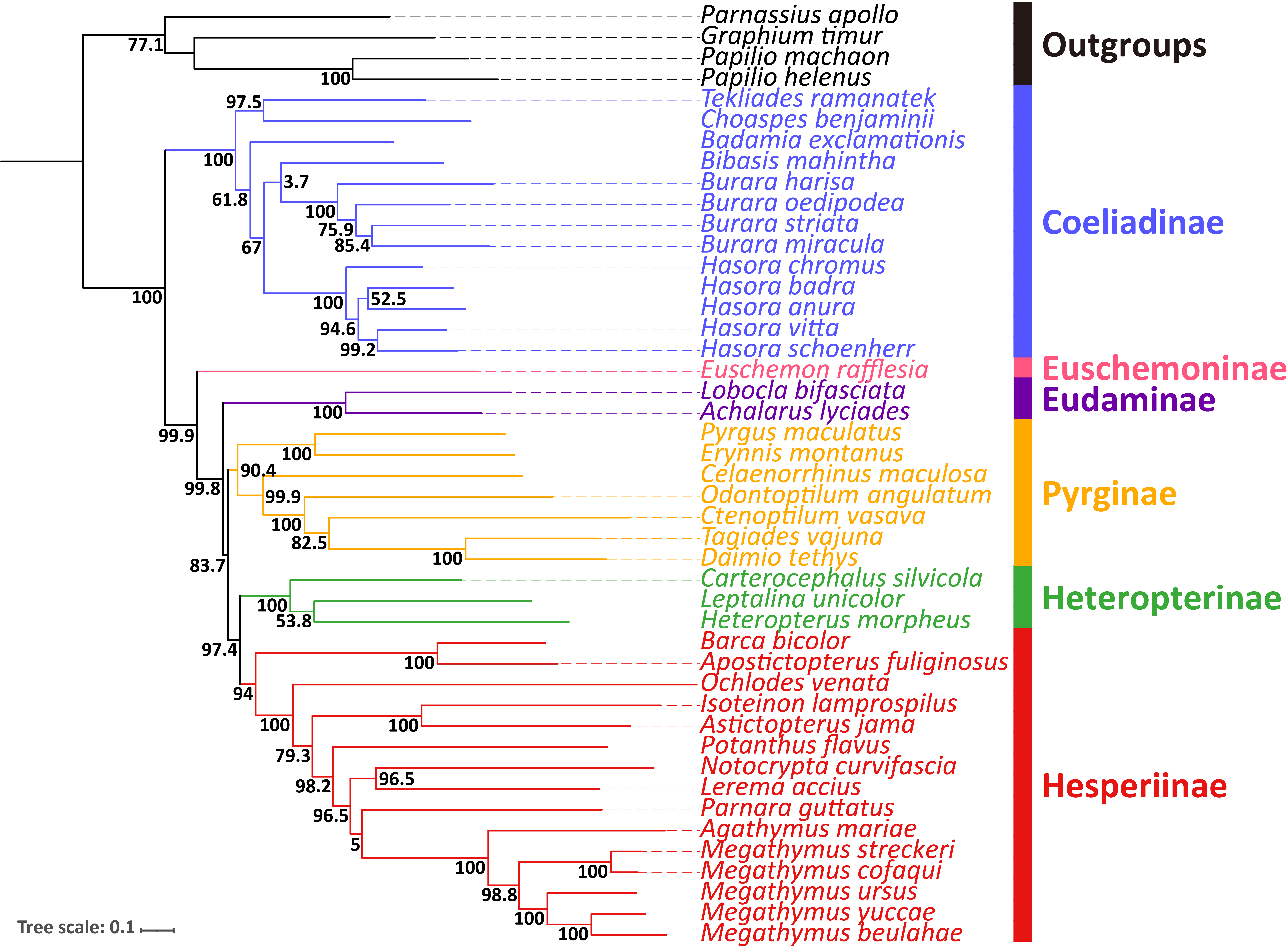

Supplement: Supplementary file 1 [file insects-12-00757-s001.zip › Figure S1.png]

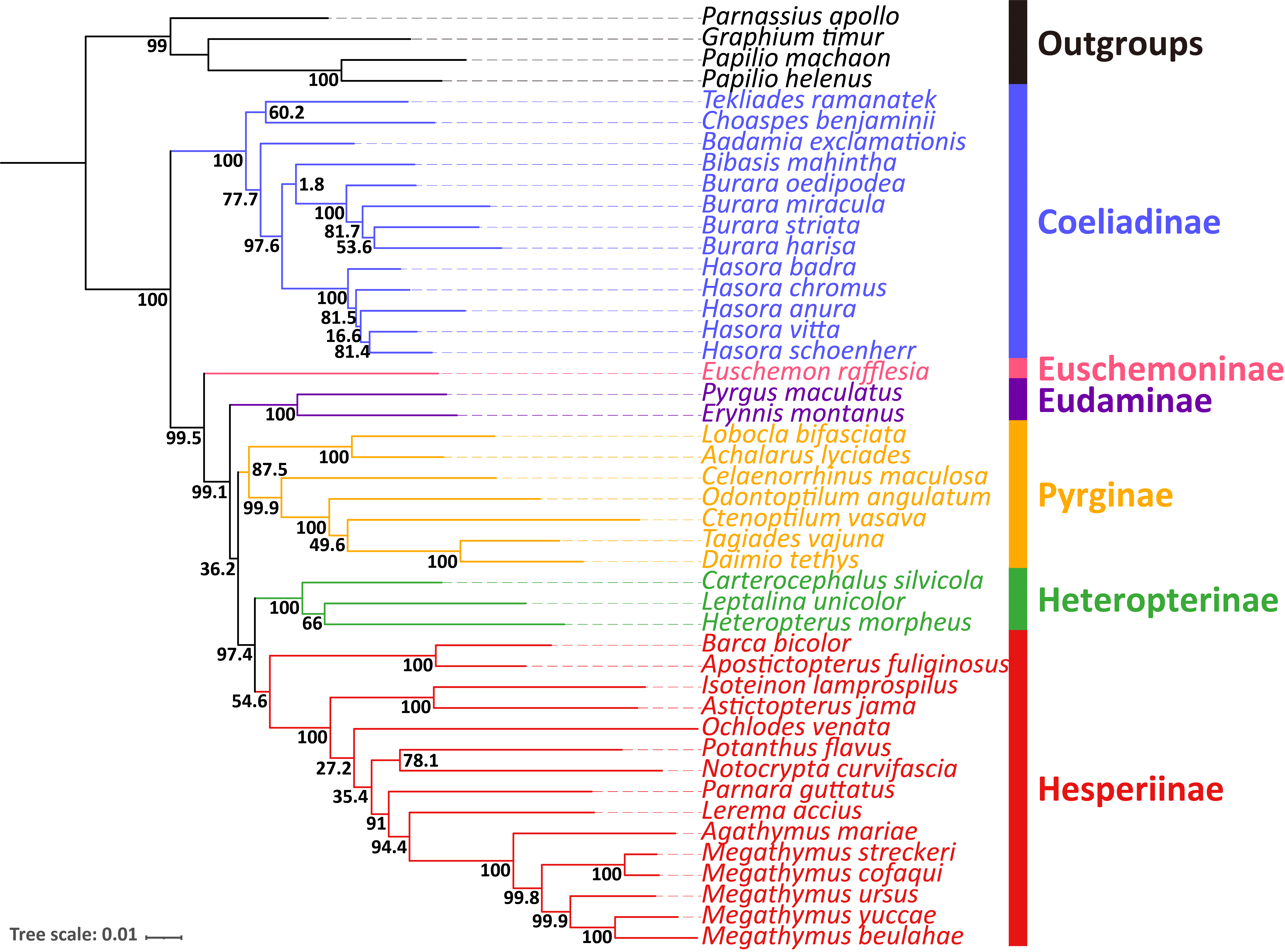

Supplement: Supplementary file 1 [file insects-12-00757-s001.zip › Figure S3.png]

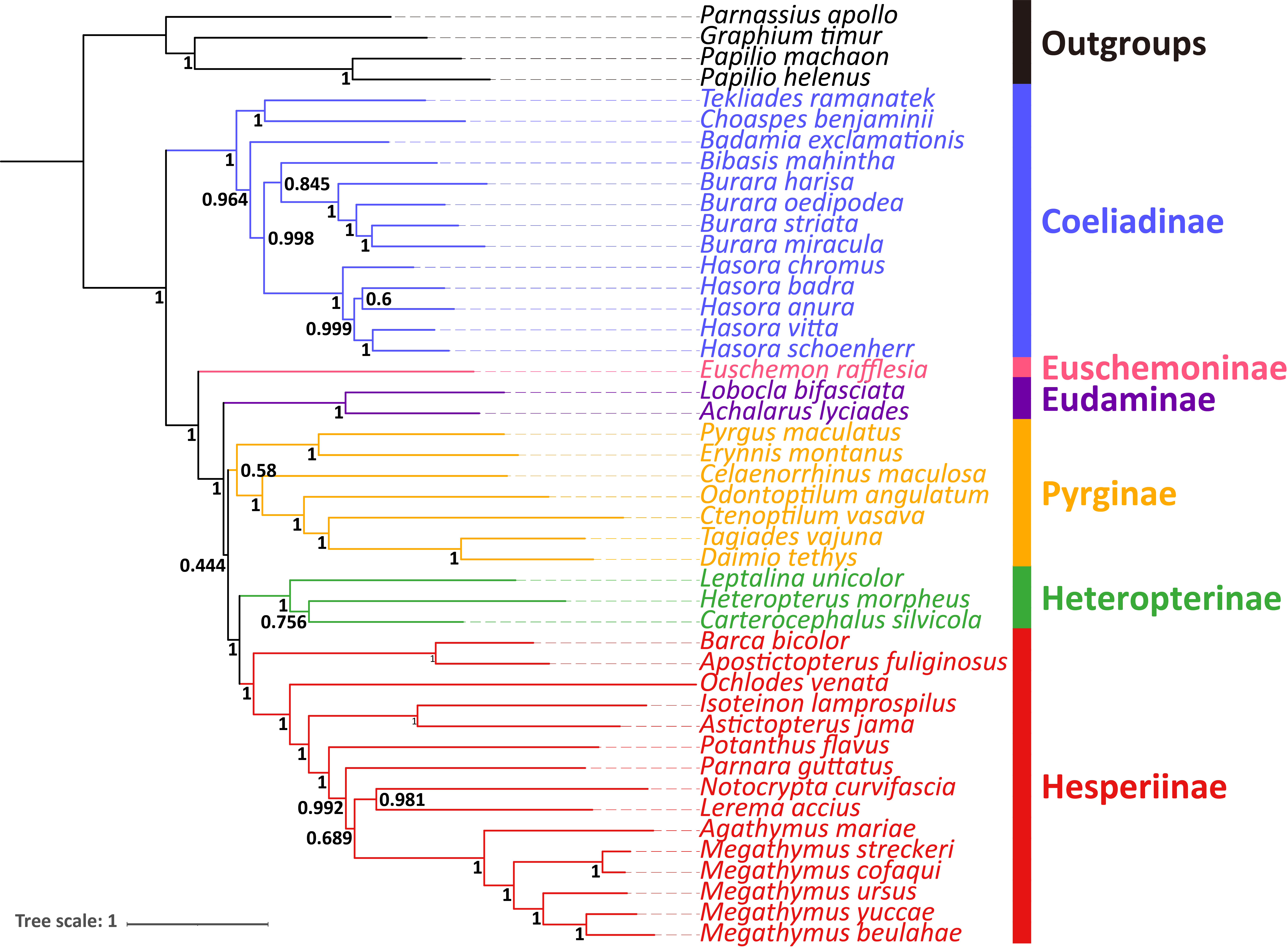

Supplement: Supplementary file 1 [file insects-12-00757-s001.zip › Figure S4.png]

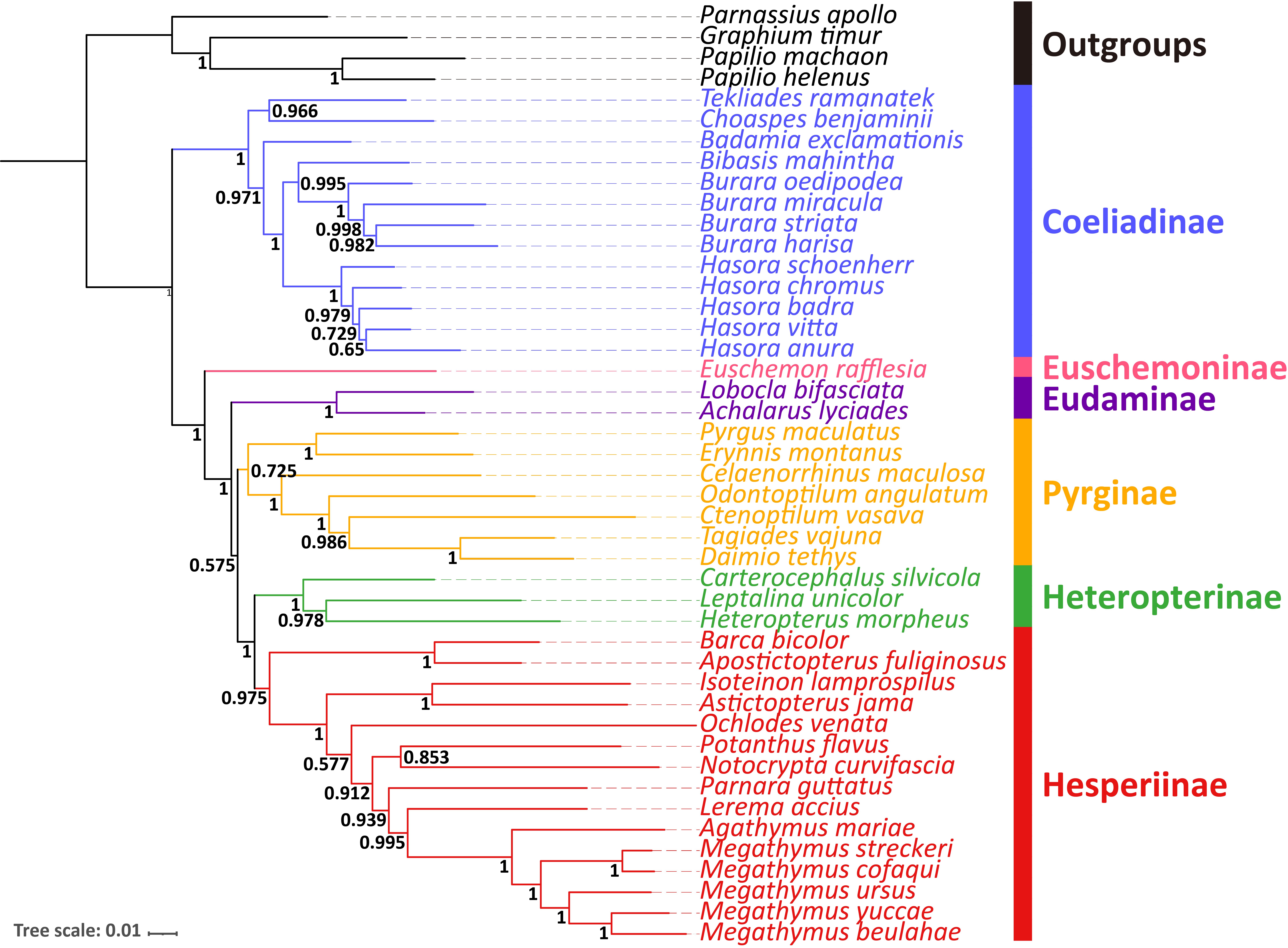

Supplement: Supplementary file 1 [file insects-12-00757-s001.zip › Figure S5.png]
